# Tafenoquine: a toxicity overview

**DOI:** 10.1080/14740338.2021.1859476

**Published:** 2021-01-04

**Authors:** Cindy S. Chu, Jimee Hwang

**Affiliations:** a Shoklo Malaria Research Unit, Faculty of Tropical Medicine, Mahidol University, Mae Sot, Thailand; b Centre for Tropical Medicine and Global Health, Nuffield Department of Medicine, University of Oxford, UK; c U.S. President’s Malaria Initiative, Malaria Branch, Division of Parasitic Diseases and Malaria, Centers for Disease Control and Prevention, Atlanta, GA, USA; d Global Health Group, University of California San Francisco, San Francisco, CA, USA

**Keywords:** 8-aminoquinoline, adverse effect, causal prophylaxis, chemoprophylaxis, radical cure, meta-analysis, plasmodium vivax, relapse prevention, drug safety, tafenoquine

## Abstract

**Introduction:**

A century-long history in 8-aminoquinolines, the only anti-malaria drug class preventing malaria relapse, has resulted in the approval of tafenoquine by the U.S. Food and Drug Administration (FDA) and the Australian Therapeutic Goods Administration (TGA) and to date registration in Brazil and Thailand. Tafenoquine is an alternative anti-relapse treatment for vivax malaria and malaria prophylaxis. It should not be given in pregnancy, during lactation of infants with glucose-6-phosphate dehydrogenase (G6PD) unknown or deficient status, and in those with G6PD deficiency or psychiatric illness.

**Areas covered:**

This systematic review assesses tafenoquine associated adverse events in English-language, human clinical trials. Meta-analysis of commonly reported adverse events was conducted and grouped by comparison arms.

**Expert opinion:**

Tafenoquine, either for radical cure or prophylaxis, is generally well tolerated in adults. There is no convincing evidence for neurologic, ophthalmic, and cardiac toxicities. Psychotic disorder which has been attributed to higher doses is a contraindication for the chemoprophylaxis indication and psychiatric illness is a warning for the radical cure indication. Pregnancy assessment and quantitative G6PD testing are required. The optimal radical curative regimen including the tafenoquine dose along with its safety for parts of Southeast Asia, South America, and Oceania needs further assessment.

## 1. Background

### 1.1. Historical background

The first 8-aminoquinoline drug synthesized was pamaquine (plasmochin) in 1926 [1]. With subsequent modifications of each 8-aminoquinoline analog from pamaquine to primaquine, the tolerability and safety have improved. Tafenoquine, a synthetic 8-aminoquinoline previously known as WR238,605, was identified as a promising antimalarial candidate in the late 1970s by the Walter Reed Army Institute of Research (WRAIR) and the Armed Forces Research Institute of Medical Sciences (AFRIMS) [2]. It underwent extensive early drug development in animal models before the first human trial was published in 1998 [2,3]. Since then, further investigations have been conducted to find safe, well-tolerated, and efficacious dosing regimens as a pre-erythrocytic agent, schizonticide, and hypnozoiticide. The early dose-finding studies for tafenoquine explored a wide range of doses, from a 4 mg to 600 mg daily dose to loading doses up to 2100 mg divided over 2 to 7 days. Over 20 human trials have been performed prior to the first regulatory approval of tafenoquine.

### 1.2. Treatment indications

Tafenoquine has received regulatory approval and clinical guidance for causal prophylaxis against malaria and *Plasmodium vivax* anti-relapse treatment (radical cure) in the USA and Australia. The US CDC guidance extended the use of radical cure tafenoquine to *P. ovale* and to presumptive anti-relapse treatment (PART) [4]. Dosing requirements range from a single 300 mg dose (for radical cure and PART) to a loading (200 mg daily for 3 days) + 200 mg weekly dose up to 6 months (for causal prophylaxis). Causal prophylaxis is recommended for persons ≥18 years old, and radical cure and PART are recommended for persons ≥16 years old. Although the label has not been updated yet, recent results from the first pharmacokinetic, safety, and efficacy analysis of tafenoquine in children ages 2–15 years with *P. vivax* malaria showed that a 50, 100, 200, and 300 mg dose based on the 4 weight bands ≥5 to ≤10 kg, >10 to ≤20 kg, >20 to ≤35 kg, and ≥35 kg, respectively, achieved the target AUC_0−∞_ with no recurrences or safety issues [5]. Tafenoquine should not be used in pregnancy (‘contraindicated’ in the Australian TGA labels and ‘not recommended’ in the US FDA labels) and during lactation if the infant is glucose-6-phosphate dehydrogenase (G6PD) unknown or deficient (contraindicated in both Australian TGA and US FDA labels). There are no published data on tafenoquine in breastmilk so lactation recommendations potentially could change pending further research (a recent lactation study found minimal excretion of primaquine in breastmilk [6]). Tafenoquine is also contraindicated when G6PD activity is decreased (<70% of the normal, specified only in the Kozenis^™^ label) or if G6PD status is unknown [7,8]. A history or current symptoms of psychosis are listed as a contraindication for the causal prophylaxis indication (Arakoda^™^ and Kodatef^®^), and as a precaution for the radical cure indication (Kozenis^™^ and Krintafel^™^). While tafenoquine does have activity against *P. vivax* gametocytes and blood-stage schizonts [9], there is no regulatory approval or dosing recommendation for transmission-blocking or blood-stage treatment of any malaria. The gametocytocidal effect of a low tafenoquine dose against *P. falciparum* is being investigated (NCT04609098). Although tafenoquine has a chemo-suppressive effect during causal prophylaxis dosing [10], it should not be used as schizonticidal monotherapy for the blood stages of *P. vivax* malaria [11].

### 1.3. Adverse effects of the 8-aminoquinolines

The 8-aminoquinoline group of drugs is known to have several toxicities that limit their use. The most serious adverse effect is a dose-dependent drug-induced hemolysis in G6PD deficient populations [12–14]; this includes tafenoquine [7]. The prevalence of G6PD deficiency is approximately 8% in malaria-endemic countries reaching 30% in some localities [15]. Mild to moderate elevations in methemoglobin levels are nearly universal after primaquine treatment, while severe, life-threatening methemoglobinemia is rare [16] only occurring in individuals with nicotinamide adenine dinucleotide phosphate (NADPH) methemoglobin reductase deficiency [17]. Abdominal pain is a dose-related adverse effect which is improved if the drug is taken after food [18]. Elevations in liver function test results are uncommon, and hepatotoxicity is rare. Early synthetic 8-aminoquinoline analogs such as pamaquine and pentaquine had significant adverse effects as noted above [19]. The subsequent development of primaquine resulted in a drug that was better tolerated and more efficacious [20]; however, low access to reliable G6PD testing and risk of drug-induced hemolysis in G6PD deficient individuals have limited the widespread use of primaquine in many *P. vivax* endemic areas. In addition, the prolonged treatment course (7 to 14 days) required for the radical cure of *P. vivax* has limited adherence [21]. Nevertheless, over 150 million doses of primaquine have been prescribed during mass drug administration programs for *P. vivax* elimination without significant numbers of passively reported severe adverse events, including hemolysis [22–26].

### 1.4. Pharmacokinetic properties and drug metabolism of tafenoquine

Unlike primaquine which reaches peak blood plasma concentrations within 4 hours, oral tafenoquine is absorbed slowly reaching peak plasma concentrations in 8–12 hours [3,27–29]. If taken after fatty food, the absorption increases by 30–40% [28,30]. The terminal plasma elimination half-life of tafenoquine is 12–16 days [27,28], which allows for a 3-day loading and weekly dose for causal prophylaxis and a single dose for PART or radical cure for malaria relapse.

Currently, tafenoquine drug metabolism studies in humans are limited thus some inference must be made from murine models. In CYP2D knockout mice, in which the equivalent P450 isoenzyme to human CYP2D6 is not expressed, tafenoquine is effective against the erythrocytic stages of *Plasmodium berghei* in both CYP2D knockout and wildtype mice [31] suggesting tafenoquine does not require CYP2D metabolism for its erythrocytic (schizonticidal) activity. In the same knockout murine model, tafenoquine is not active against *P. berghei* sporozoite inoculations, which suggests CYP2D metabolism is required for tafenoquine’s exoerythrocytic (liver schizont and hypnozoite) activity [32]. However, in humans *P. vivax* recurrence after tafenoquine is not associated with the intermediate CYP2D6 phenotype (by activity score assignment), which suggests CYP2D6 is not required for radical cure [33]. The contrasting results from murine and human studies indicate that more research in humans is needed to understand the metabolism of tafenoquine.

### 1.5. Drug interactions

As the metabolic pathway of tafenoquine is not yet fully identified, the evaluation of drug interactions has been limited to its potential schizonticidal partner drugs. Chloroquine in combination with tafenoquine is not associated with persistent changes in tafenoquine blood plasma concentrations or clinically significant adverse effects when compared to tafenoquine alone [34]. When dihydroartemisinin-piperaquine is co-administered with tafenoquine, the maximal blood plasma concentrations of tafenoquine are raised and the time to maximal concentration is faster, but the overall drug exposure is similar to tafenoquine monotherapy [35]. Artemether-lumefantrine co-administration has no effect on tafenoquine blood plasma concentrations [35]. In early 2020, GlaxoSmithKline released a notification (in the form of a Dear Healthcare Provider letter) that Krintafel^™^ (the branded form of tafenoquine in the USA) should be combined only with chloroquine and not with other antimalarials, e.g. artemisinin-based combination therapies (ACT) [36]. This announcement was prompted by low relapse-free rates of the single-dose tafenoquine combination with dihydroartemisinin-piperaquine (21%) similar to dihydroartemisinin-piperaquine alone (11%) in Indonesian soldiers returning to non-endemic areas and was not due to issues with safety [37]. More investigation is needed to assess drug–drug interactions and pharmacodynamics with tafenoquine, especially in areas with chloroquine resistance and/or in countries where ACTs are the nationally recommended treatment for all malaria.

### 1.6. Rationale

Tafenoquine has a similar side effect profile to that of primaquine, which means generally it is well tolerated. However, tafenoquine causes clinically significant elevations in methemoglobin levels in some individuals (with the ≥400 mg daily doses used during the dose-finding trials) and hemolysis in persons with intermediate and deficient G6PD activity. Quantitative G6PD testing is needed so that tafenoquine is restricted to individuals with >70% G6PD activity, as due to a long plasma terminal elimination half-life, it cannot be stopped if a severe adverse event occurs. This systematic review analyzes the adverse events of tafenoquine that have occurred in humans in the literature to date and attempts to present them in a clinically useful way.

## 2. Methods

A search of English-language articles available in Embase, Ovid Medline, Scopus, Cochrane Library, CINAHL, Global Health (CABDirect), clinicaltrials.gov, and WHO International Trials Registry was conducted on 25 June 2020, using the terms ‘tafenoquine OR arakoda OR krintafel OR kodatef OR kozenis OR WR238605 OR WR-238605.’ The references from review articles and meta-analyses and the FDA labeling for both Krintafel^™^ and Arakoda^™^ were also reviewed to identify any additional studies. Randomized controlled trials (RCTs), quasi-experimental studies, and randomized cross-over trials administering tafenoquine in any dosing to human subjects were included in the review. Data on study characteristics including dosing regimen, outcomes, and adverse events were extracted. For studies with a comparison arm (placebo or another antimalarial), the risk ratios (also called relative risk (RR)) with corresponding 95% confidence intervals (CIs) for adverse event outcomes were grouped by the drug regimen for comparison. If the study included more than one tafenoquine dosing regimen, only the dosing regimen closest to the current radical cure or prophylaxis indication dose was included in the meta-analysis. We inspected forest plots for overlapping CIs and assessed statistical heterogeneity in each meta-analysis using the I2 statistic and Chi2 test. A fixed-effect meta-analysis was conducted if heterogeneity was absent; otherwise, a random-effect model was used due to consideration of dosing and methodological heterogeneity in these outcomes. Overall summary estimates were not included if there was significant heterogeneity.

**Figure 1.**
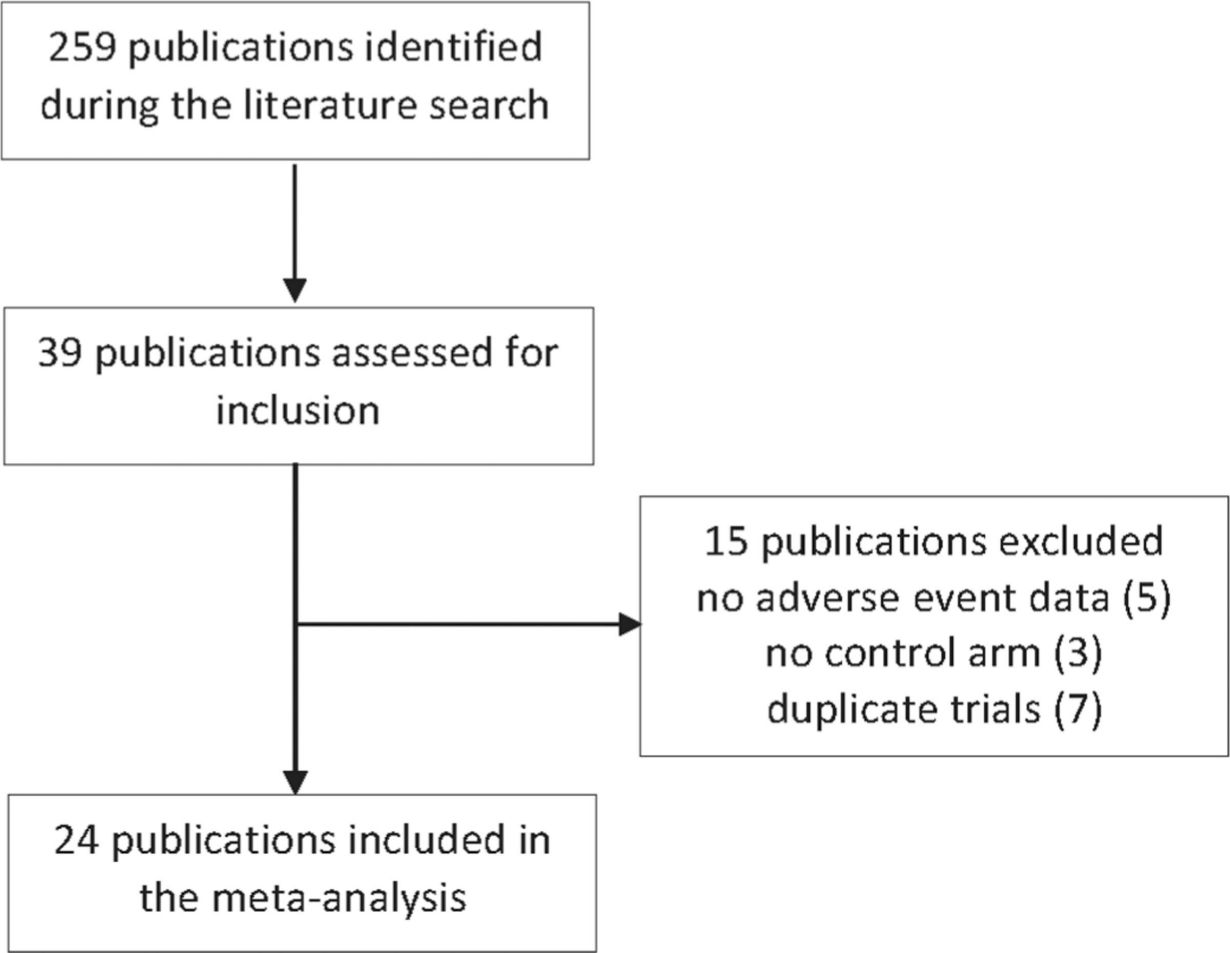
Selection of publications.

The search resulted in 259 publications of which full manuscript review was conducted for 39 articles to assess for inclusion. Fifteen studies were excluded from the meta-analysis (five contained no adverse events data; three did not include a non-tafenoquine comparison arm; seven studies did not present unique results). Twenty-four studies contributed at least one unique adverse event data for the meta-analyses. Figure 1.

## 3. Neuropsychiatric adverse effects

**Figure 2.**
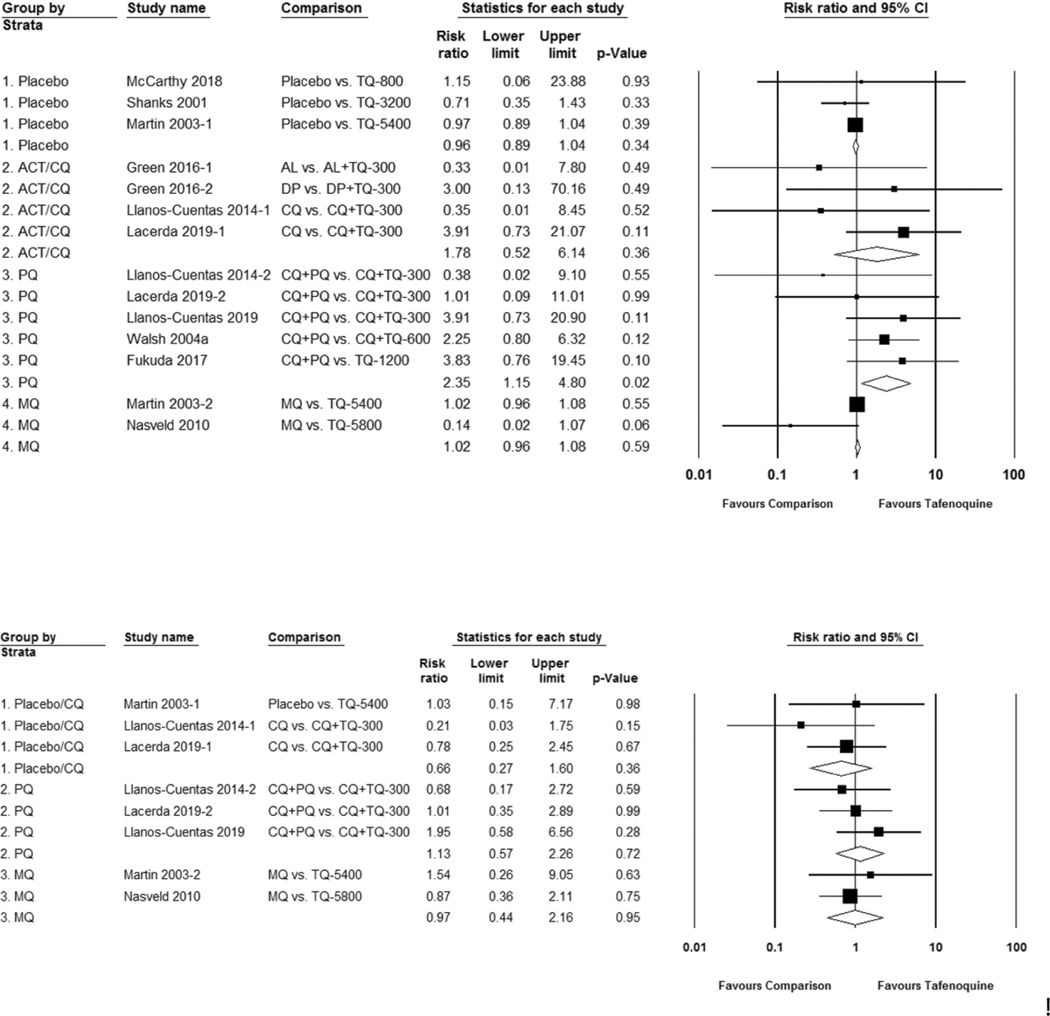
Risk ratio of neuropsychiatric symptoms with tafenoquine as compared to placebo or a control group. Figure 2(a). Any reported neurological adverse effect. (b). Any reported psychiatric adverse effect.

The neuropsychiatric effects caused by mefloquine, a quinoline-methanol derivative, are well known though they are less commonly associated with 8-aminoquinolines. Data on symptoms most commonly reported (data collection may have been passive or active) with tafenoquine are headache, dizziness (vertigo), and lethargy (weakness, fatigue, somnolence) [38]. Supplement Figure 1–3. Overall, neurological symptoms did not occur more commonly after tafenoquine dosing. Primaquine compared to tafenoquine was associated with a significantly higher risk of any reported neurologic symptom; RR 2.35 (95% CI 1.15 to 4.80). Figure 2(a). Data on neuropsychiatric symptoms such as abnormal dreams, tremors, and effects on coordination, mood, and memory were less commonly collected; detailed psychiatric data were found in five published clinical trials [39–43]. Compared to placebo or chloroquine, primaquine, and mefloquine, tafenoquine was not associated with an increased risk of psychiatric symptoms; RR 0.66 (95% CI 0.27 to 1.60), RR 1.13 (95% CI 0.57 to 2.26), and RR 0.97 (95% CI 0.44 to 2.16), respectively. Figure 2(b). The US FDA Adverse Events Reporting System contains six psychiatric serious adverse events including two suicides associated with the post-market prophylaxis indication [44]. Other neuropsychiatric adverse events (e.g. headache, dizziness, and lethargy/weakness/fatigue) were similar between tafenoquine and placebo or the comparator drug group. Supplement Table 1A-C.

Data collection on neurologic symptoms has not been systematic and active questioning for psychiatric symptoms has not been performed routinely in or stated within the methods across the tafenoquine clinical trials reviewed. Careful follow-up and patient counseling on potential psychiatric adverse effects, especially with the causal prophylaxis indication, should be monitored carefully in these populations. Tafenoquine, as Arakoda^™^ and Kodatef^®^ for causal prophylaxis, is contraindicated in persons with current or history of psychotic disorder or symptoms. With the single-dose regimens for the radical cure, Krintafel^™^ and Kozenis^™^, a current or history of psychiatric illness, whether serious or not, is listed as warnings and precautions for use, so should be avoided.

## 4. Ophthalmologic adverse effects

The chemical structure of tafenoquine is similar to other cationic amphophilic drugs; one example of such an agent is chloroquine. This group of drugs causes a dose-dependent increase in intracellular phospholipid content resulting in excessive accumulation in tissues [45]. Excess phospholipid accumulation in the eye causes corneal epithelial deposits [46], described as vortex keratopathy because of the characteristic whorl or verticillate patterns. Vortex keratopathy is typically benign and does not cause symptoms [47]. There are currently five published studies where fundoscopic and retinal examination, visual field and acuity tests, and color vision were assessed after tafenoquine was given [39,41,42,48,49]. All studies except one have baseline measurements [42]. Table 1 and Supplement Table 1B. Additionally, in one pharmacokinetic study, ophthalmologic assessments showed a trend of declining visual acuity in the tafenoquine-treated groups (TQ450mg daily × 2 days with and without chloroquine), but the trial was not powered for ophthalmic comparisons between groups [34]. Overall, the data suggest that the development of tafenoquine induced vortex keratopathy is dose dependent. Corneal findings are reversible and even at the greatest tafenoquine exposure (200 mg loading dose plus 200 mg weekly for 26 weeks) vortex keratopathy resolves, up to 1 year after causal prophylaxis completion [42]. In all studies that assessed vision, the clinical effects (changes in visual acuity, color vision, adverse effects) were mild and no different than the comparator. In three out of five studies, the comparator was chloroquine which, as stated above, also causes vortex keratopathy. These data indicate that tafenoquine causes dose-dependent reversible changes in the ocular tissue that generally do not affect vision. With prolonged dosing regimens such as for causal prophylaxis, patients should be advised to seek ophthalmologic assessment if they have vision changes. Single-dose regimens are unlikely to cause pathologic changes in the eye. Health-care providers and patients should be aware that the product label for tafenoquine limits causal prophylaxis dosing to 6 month duration.

## 5. Cardiac adverse effects

Some drugs in the quinoline class of antimalarials block the cellular influx of sodium or potassium ions during cardiac repolarization [50]. This results in a prolongation of the QT interval. If the corrected QT (QTc) interval is greater than 500 ms there is a propensity to develop torsades de pointes, a potentially fatal dysrhythmia. Chloroquine, piperaquine, and amodiaquine [51], which are 4-aminoquinolines, can cause a prolonged QTc interval. However, when given in the recommended therapeutic doses for malaria treatment, the risk for sudden unexplained death is not greater than in the general population [52,53]. In laboratory and animal models, primaquine has been shown to inhibit the cardiac sodium and potassium channels [54,55] at molecular concentrations greater than approximately 96-fold the equivalent plasma concentration after a 15 mg daily dose in humans [54]. The clinical effects of primaquine on the QT interval are not well studied, though current evidence suggests that in usual antimalarial doses primaquine does not cause a prolonged QTc interval.

In contrast to primaquine, the clinical electrocardiographic (ECG) changes with tafenoquine have been well evaluated. Supplement Table 1C. In 9 studies a variety of doses have been assessed, in dose-ranging studies from a 4 mg single dose to a 1200 mg loading dose divided over 3 days, to a 600 mg loading dose divided over 3 days that is continued weekly for causal prophylaxis [3,34,35,39–42,56]. Tafenoquine in addition to an ACT or CQ, or compared to primaquine, did not cause study defined ECG changes at the single radical cure dose (RR 1.72 95% CI 0.80 to 3.72) [3,34,35,42,56]. Supplement Figure 4. Although not statistically significant, ECG changes were more likely to occur with the ACTs prolonging QTc interval or chloroquine alone (RR 2.37 95% CI 0.77 to 7.29) [35,39,40]. Typically, ECG changes were defined as QTc >480 ms with or without a > 60 ms increase from baseline. No publications were found that investigated the effect of tafenoquine on cardiac ion channel inhibition. Based on currently available evidence, tafenoquine does not cause clinically significant ECG changes nor are there reports of sudden unexplained death associated with its use. Thus, pre-dose ECG screening is not indicated if there are no risk factors for prolonged QT such as unexplained syncope, concomitant administration of drugs that prolong QT interval (e.g. negative chronotropic agents, amitriptyline, azithromycin, etc.), or a family history suggestive of a congenital prolonged QT syndrome (e.g. unexplained death).

## 6. Gastrointestinal adverse effects

**Figure 3.**
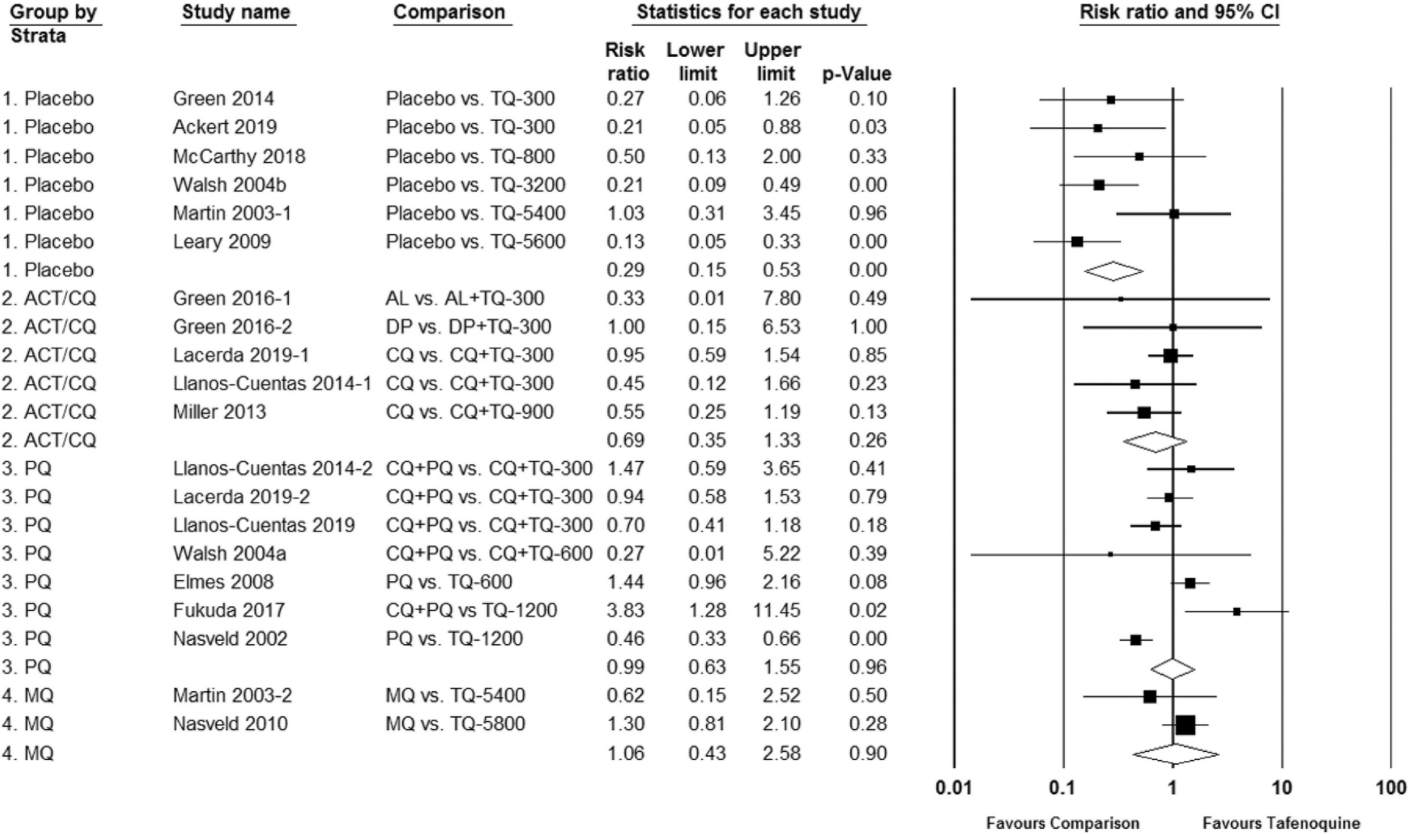
Risk ratio of nausea or vomiting with tafenoquine as compared to placebo or control group.

As with other 8-aminoquinolines, abdominal pain is a common adverse effect after ingesting tafenoquine and appears to be dose dependent. Supplement Table 1A and Supplement Figure 5. The mechanism of action is unknown in humans. In animal studies, mucoid degeneration of the gastric cavity has been observed microscopically after primaquine administration in dogs, but not monkeys or rats [57]. Nausea or vomiting is observed to occur more frequently with tafenoquine when compared to placebo; RR 0.29 (95% CI 0.15 to 0.53). Figure 3. These symptoms may be dose dependent. A dose-dependent effect can also be seen with diarrhea, but the risk compared to placebo was not statistically significant; RR 0.75 (95% CI 0.45 to 1.24). Supplement Table 1A, Supplement Figure 6. Elevations in alanine transferase (ALT) are no different between tafenoquine and placebo or a comparator drug [58], Supplement Table 1C, and there have been no published reports of acute hepatic failure associated with tafenoquine use to date. Abdominal pain is alleviated if the tafenoquine dose is taken after a meal [3,8,59].

## 7. Hematologic adverse effects

No clinically significant effects on the bone marrow have been observed on complete blood counts with tafenoquine. However, the most well-known hematologic adverse effect of the 8-aminoquinolines has been the dose-dependent hemolysis observed in G6PD deficient individuals with decreased G6PD activities. G6PD deficiency is inherited in a sex-linked pattern and occurs most commonly in malaria-endemic regions [60]. There are over 200 G6PD mutations (and over 400 G6PD variants) identified [61], which determine the severity of the residual G6PD activity. Severe mutations (currently classified as Class II variants [62]) cause very low G6PD activity where the reticulocytes are also deficient so hematologic recovery may be insufficient [63]. In mild mutations (currently classified as Class III variants [62]) the G6PD activity in reticulocytes is high enough (or nearly normal) so that hematologic recovery is sustained even if the hemolytic agent is continued [64]. Hemizygous G6PD mutated males are G6PD deficient and correspondingly will have a deficient (abnormal) result on a qualitative G6PD test. Females may be either homozygous or heterozygous for a G6PD mutation. Homozygous females will also have G6PD deficiency and a corresponding deficient G6PD qualitative test. Heterozygous females undergo random X chromosome inactivation (lyonization) during early embryogenesis and the same pattern of inactivation is then maintained in the red blood cells. This means that G6PD activity in heterozygous females can range from very low to normal; for the females with G6PD activity in the range of intermediate to normal (~30–70%), a qualitative G6PD test with a common threshold for deficiency at <30% activity (e.g. fluorescent spot test or currently available lateral flow rapid diagnostic tests) will give a normal result. Heterozygous females with G6PD Mahidol (reticulocytes have normal enzymatic activity in Mahidol variant [65]) and intermediate G6PD activity can generally tolerate a low (15 mg adult dose or 0.25 mg/kg day for 14 days) or high (30 mg adult dose or 0.5 mg/kg daily for 14 days) dose primaquine regimen [66]. If severe hemolysis occurs (not uncommon if there is a concomitant *P. vivax* infection), primaquine can be stopped. However, as tafenoquine has a long plasma elimination half-life, it cannot be stopped after a dose is given if clinically relevant hemolysis occurs. Thus, for safety reasons, tafenoquine is only given when the G6PD activity is >70% of the male population median [7]. To identify intermediate G6PD activity, a quantitative or semi-quantitative phenotypic G6PD test (e.g., spectrophotometry or biosensors) is required. These tests give a numeric result which is then categorized as normal, intermediate, or deficient and allow for safer 8-aminoquinoline prescription in all patients, irrespective of sex. New point of care qualitative and quantitative G6PD diagnostics are available but need further evaluation of their effectiveness and feasibility at the patient level, sex-specific considerations, and appropriate delivery models for implementation.

**Figure 4.**
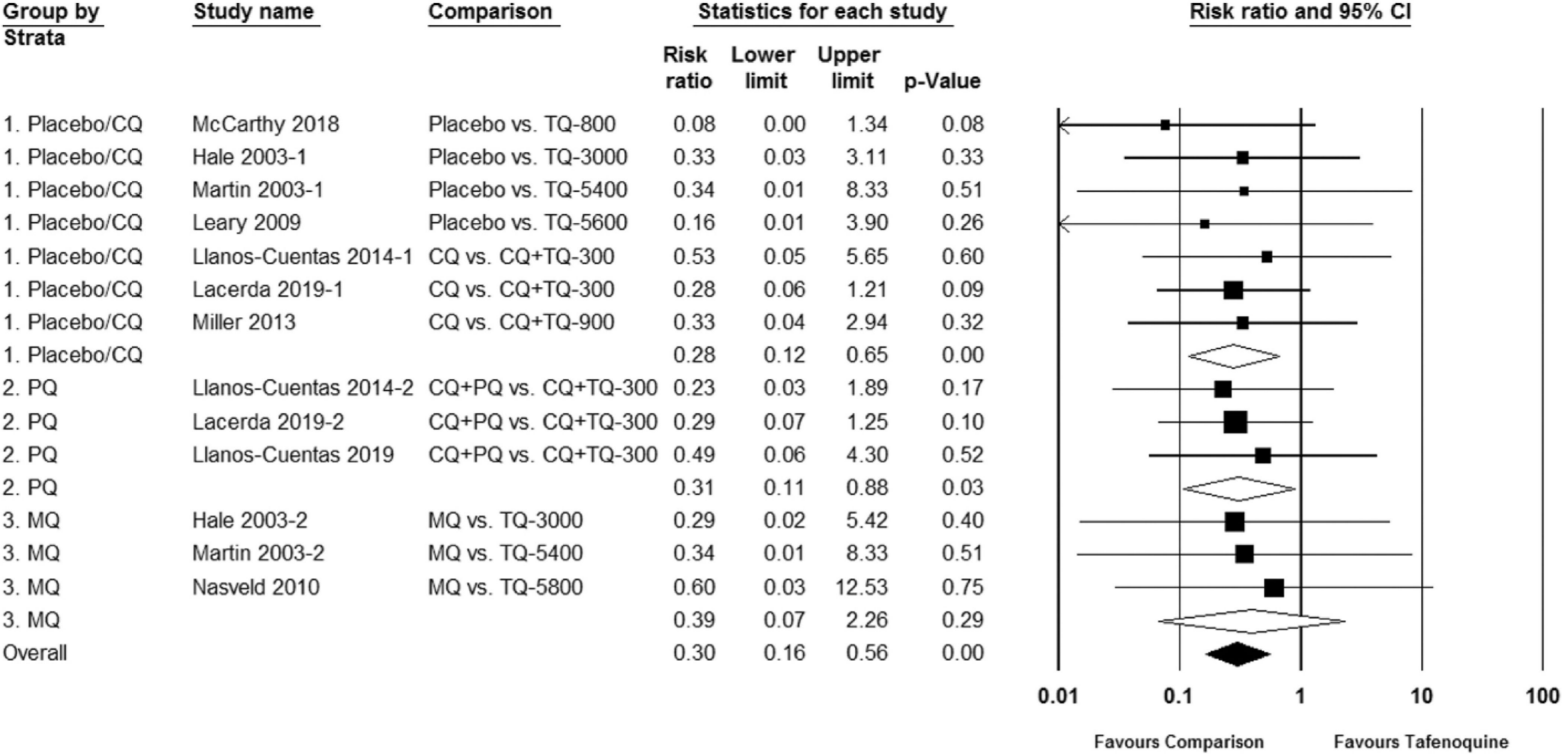
Risk ratio of hemolysis in G6PD-normal subjects after tafenoquine as compared to placebo or control group.

In the studies reviewed, a total of 20 G6PD heterozygous females received tafenoquine. One safety trial intentionally included females with intermediate G6PD deficiency. In this trial, 17 G6PD Mahidol-variant heterozygous females with G6PD activity ranging from 40% to 60% received either standard low-dose primaquine 15 mg daily for 14 days or a 300 mg single dose of tafenoquine for radical cure. Similar hemoglobin reductions and recovery were observed between the two groups [7]. When the study’s dose-limiting toxicity threshold was reached (≥3/6 subjects with ≥2.5 mg/dL hemoglobin or ≥7.5% hematocrit reduction from baseline) at 300 mg, the tafenoquine dose was not escalated further. Excluding the above trial with G6PD intermediate females, in this meta-analysis the risk ratio of study-defined hemolysis favored placebo or chloroquine co-administration (RR 0.28 95% CI 0.12 to 0.65) and primaquine (RR 0.31 95% CI 0.11 to 0.88), but was not statistically significant for mefloquine (RR 0.39 95% CI 0.07 to 2.26) when compared to tafenoquine. Figure 4. The studies included in the meta-analysis vary in 1) vivax patients versus healthy volunteers, 2) assessment of G6PD status and study inclusion using qualitative versus quantitative G6PD tests, and 3) the day of follow up which influences the moment at which hemoglobin/hematocrit is at its nadir. These differences confound the adverse event reporting and hemolysis assessment such that a meta-analysis as a dichotomous outcome may not be the ideal method for analyzing hemolytic risk.

No serious adverse events related to hemolysis have occurred in G6PD normal individuals with >70% activity; however, in a malaria prophylaxis, clinical efficacy trial two females with G6PD deficiency were misclassified as G6PD normal. Both developed large hemoglobin reductions after a loading dose with 400 mg tafenoquine daily for 3 days; one was asymptomatic and the other required blood transfusions [8]. Higher single doses of tafenoquine may be necessary for an efficacious radical cure in parts of SE Asia [41]; doses greater than a single 300 mg dose have not been assessed in individuals with intermediate G6PD activity. Though the safety trial in 17 G6PD Mahidol heterozygous females with intermediate G6PD activity [7] showed a similar moderate hemolysis and subsequent recovery in low dose (15 mg adult dose) 14-day primaquine and 300 mg single dose tafenoquine, pre-defined study safety measures halted further assessment of higher tafenoquine doses. If higher anti-relapse tafenoquine doses are evaluated in persons with intermediate G6PD activity, understanding the relationship of G6PD variant and quantitative G6PD activity with the degree of the hemolytic and recovery responses will be needed.

Elevations in methemoglobin in clinical trials have been reported as means (standard deviation), medians (range), absolute or mean maximal values, or mean changes from baseline (confidence intervals) which makes comparison across studies and dosing regimens difficult. Nonetheless, it can be observed that tafenoquine is associated with methemoglobin elevation and that similar to primaquine, it appears to be dose dependent. Overall, the elevations with the currently approved dosing regimens for causal prophylaxis and radical cure are mild and less pronounced than with primaquine [7,11,34,39–41]. Supplement Table 2. However, maximal absolute methemoglobin values in some trials indicate that there are individuals who do develop clinically significant elevations [11,39,56,59,67].

Even when reliable G6PD testing is available, patients should be advised of signs and symptoms of hemolysis to ensure that symptoms of hemolysis are promptly recognized, and appropriate treatment is provided in case of unexpected hemolysis (e.g. misdiagnosed G6PD deficient individuals). Moreover, the signs and symptoms of methemoglobinemia (e.g., cyanosis, headache, dizziness, shortness of breath) should be considered post administration.

## 8. Dermatologic adverse effects

**Figure 5.**
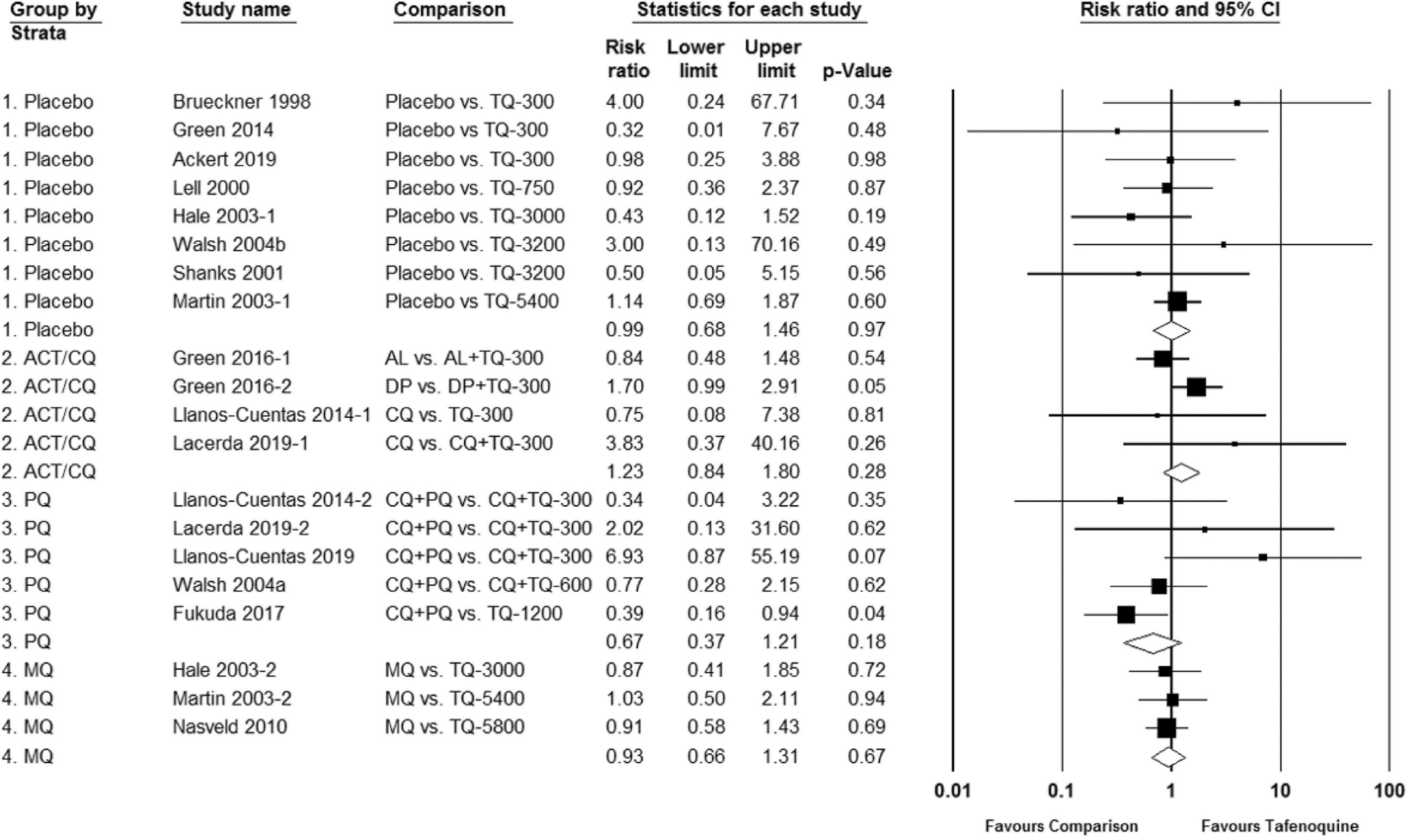
Risk ratio of any dermatologic symptoms after tafenoquine as compared to placebo or a comparator.

The quinoline derivatives have had varying associations with dermatologic adverse effects. Pruritis and maculopapular rash have been reported with mefloquine, chloroquine, and quinine, though pruritis is a common complaint with chloroquine in populations with more melanin content in the skin [68]. Chloroquine is known to bind to melanin [69,70]. Rarer are reports of photosensitivity, urticaria, vasculitis, lichen planus, and severe mucocutaneous rash. Skin manifestations resulting from primaquine are rarely reported. Dermatologic symptoms do not appear to be significantly associated with tafenoquine regardless of the comparison arm. Figure 5.

## 9. Summary

Tafenoquine has been approved for malaria prophylaxis and radical cure of *P. vivax* in the USA and Australia. To date, tafenoquine has been registered for the radical cure indication in Brazil and Thailand. Tafenoquine appears to be well tolerated in eligible populations. The common adverse effects (ophthalmologic, gastrointestinal, and hematologic) are dose dependent; thus, the single dose currently indicated for radical cure and PART is unlikely to cause severe symptoms. Consistent with other systematic reviews [38], this meta-analysis shows that neuropsychiatric symptoms do not occur more frequently with single-dose tafenoquine when compared with primaquine or no radical cure. Because psychiatric data are not collected systematically across studies and not always by active questioning, this relationship should continue to be monitored closely through post-market surveillance. Long-term courses of tafenoquine for causal prophylaxis should not be prescribed to persons with preexisting or current psychosis. For the radical cure indications, caution should be taken during treatment if there are preexisting or current symptoms of any psychiatric illness, however mild. Vortex keratopathy may occur but does not typically result in vision changes. If there are visual symptoms, tafenoquine should be stopped. The pathologic findings of vortex keratopathy are reversible even at higher doses. Based on current evidence, tafenoquine does not appear to prolong the QT interval. Nevertheless, the tafenoquine regimen for causal prophylaxis should be avoided in persons with a history of unexplained syncope, a family history that suggests a risk for prolonged QT (unexplained death, known congenital long QT syndrome). Gastrointestinal symptoms can be alleviated by taking food prior to dosing but still may occur as this precaution was taken in nearly all the studies in this systematic review. Tafenoquine is contraindicated in G6PD intermediate and deficient individuals so clinically significant non-malarial hemolysis is unlikely to occur if quantitative G6PD testing is performed. Elevations in methemoglobin overall are mild; however, methemoglobin elevation may be clinically significant in some individuals, especially in the presence of NADPH methemoglobin reductase deficiency. Dermatologic adverse effects are similar between tafenoquine and placebo.

Although the safety of mass primaquine administration is well documented (with clinical monitoring as reported) [25,71], hemolysis remains a major concern among managers of national malaria programs [72]. In contrast to tafenoquine, primaquine can be stopped if hemolysis is detected. This offsets the benefit of weekly tafenoquine dosing for causal prophylaxis and a single tafenoquine dose for PART or radical cure. Evidence suggests that in parts of SE Asia and Oceania (where the short-latency *P. vivax* phenotype is prevalent) higher doses of primaquine (total dose 7 mg/kg) are needed for radical cure [73–75]***. This may also be the case with tafenoquine as the radical curative rate with the 300 mg single dose was 19% lower than supervised low dose primaquine (15 mg daily for 14 days) in the meta-analysis of SE Asian sites (the DETECTIVE and GATHER trials) and though the South American meta-analysis showed no difference between the same study arms, the approximately 65% radical curative rate was similarly low in both groups suggesting low cure rates for both 8-aminoquinoline regimens or high reinfection rates [41]. Suboptimal tafenoquine dosing for radical cure results in low relapse prevention rates that contribute to persistent *P. vivax* transmission and the burden of recurrent *P. vivax* malaria infections with associated morbidities (e.g. anemia, hospitalization, low birth weight). The challenge is to balance the toxicity of tafenoquine with the benefit of efficacious radical cure.

The main limitation of this review is that significant study heterogeneity existed across these studies in design, objective, and outcome evaluation. For example, drug regimens may have been supervised differently between studies which could affect adherence and the occurrence of adverse events in comparator drug groups. There was some variability in adverse events reporting terminology requiring the reviewers to use clinical judgment to combine corresponding symptoms and diagnoses such as diarrhea and gastroenteritis. The hemolysis meta-analysis could not provide clinically meaningful results because of the variability between study inclusion criteria and follow up frequency. Additionally, the descriptive statistics for methemoglobin results varied widely and could not be combined for a meta-analysis. However, the study methods and data presentation were less disparate than expected overall.

In conclusion, tafenoquine is generally well tolerated. There is no convincing evidence for neuropsychiatric, ophthalmic, dermatologic, and cardiac toxicities although precautions have been advised for psychiatric adverse events for the prophylaxis indication. Tafenoquine must not be given in pregnancy, during lactation of G6PD unknown or deficient infants, and when G6PD activity is <70%, quantitative G6PD testing is required. In some situations, health-care providers may choose to perform additional pre-screening history and examination before drug administration. In this case, a systems review could include active questioning on the psychiatric, ocular, cardiac, and hematologic systems. Other pre-treatment investigations could be performed based on the identification of risk factors during pre-screening history and examination. However, universal pre-screening may not be practical and there is no evidence to suggest that it is needed for the radical cure dose in low-resource settings or within large-scale malaria programs, except for pregnancy and G6PD status. Most adverse events with tafenoquine occur early (within the first 2 weeks of the dose). Although less likely, symptoms might be related to tafenoquine if they are detected up to 3 months after the last dose, representing five plasma elimination half-lives of tafenoquine. Health-care worker training and patient awareness of potential adverse effects and the timing of the effects, thresholds for referral, and management options are important for the safe deployment of tafenoquine.

## 10. Expert opinion

The therapeutic tools to eliminate malaria infections are available but need extensive implementation if elimination is to be achieved quickly. Tafenoquine is a generally well-tolerated drug which makes it a good alternative for causal prophylaxis, alongside atovaquone-proguanil and primaquine, and for a radical cure, alongside primaquine. The single dosing regimen gives tafenoquine a substantial advantage over 7 or 14-day primaquine regimens for a radical cure; providing national malaria programs a simpler cost-effective drug that can reduce malaria relapses. However, the interaction between 8-aminoquinolines and G6PD deficiency is a major obstacle to the safe deployment of tafenoquine. Tafenoquine cannot be ‘stopped’ if clinically relevant hemolysis is detected after a dose. Other uncommon adverse events can have profound effects in some individuals (e.g. methemoglobinemia, neuropsychiatric effects with the prophylaxis dosing). Moreover, the optimal tafenoquine dose for relapse prevention in parts of SE Asia, Oceania, and South America remains unresolved. The role of tafenoquine for all use scenarios is unclear without easy to use point of care quantitative or semi-quantitative G6PD testing, a means for detecting clinically relevant hemolysis (mainly at lower levels of health delivery), and if hemolysis is detected, ready access to higher levels of health-care services.

Although this review focused on the safety and tolerability of tafenoquine, outstanding questions remain about the role of tafenoquine in malaria case management, prevention, and elimination. In malaria-endemic parts of SE Asia, South America, and Oceania, the efficacy of higher single doses of tafenoquine should be compared to the currently recommended 300 mg dose so that relapse prevention can be optimized. In addition, more studies assessing schizonticidal combinations with tafenoquine other than chloroquine are needed especially in areas with chloroquine resistant *P. vivax*. Formal recommendations for tafenoquine radical cure dosing in children <16 years old are anticipated and will need continued evaluation.

Current efforts toward operational research primarily focus on the delivery of treatment such as the ease, frequency and cost of G6PD testing at different health-care levels. Provision of genetic counseling and documentation of G6PD status, ideal approaches to detecting hemolysis by rural community health workers or volunteers, adherence to treatment regimens, and further clinical trial comparisons between tafenoquine (for persons of all ages) and primaquine are still needed for assessing the reliability of G6PD tests, treatment efficacy and effectiveness, quality of adverse event monitoring, and community acceptance. As malaria elimination approaches reality, determining the role of and appropriate targeting of tafenoquine and primaquine in elimination interventions (mass drug administration, targeted malaria elimination, active case detection, etc.) will be essential in accelerating malaria elimination.

In addition to field-based and comparative clinical trials, more understanding can be gained in the pharmacokinetics and pharmacodynamics of 8-aminoquinolines in the blood and breastmilk. The relationship of the cytochrome P450 isoenzyme CYP2D6 to the drug metabolism of tafenoquine in humans has not been ascertained clearly yet. If higher doses of tafenoquine are needed for radical cure, its effect on hemolysis and hematologic recovery in G6PD intermediate females with different G6PD variants should be determined. This emphasizes the importance of sustaining laboratory-based research in tafenoquine (and primaquine) even as global rates of malaria infections decline.

In the next several years, safe implementation of tafenoquine (with G6PD testing) in malaria-endemic settings for the efficacious prevention of relapse should be established at primary health-care levels by qualified health-care providers. It can then be determined if G6PD testing and tafenoquine treatment can be provided at lower levels of health care by community health workers where tafenoquine can expand its role as a major therapeutic in vivax malaria elimination.

## Supplementary Material

Supplement

## Figures and Tables

**Table 1.** Observations of ocular and visual changes after dosing with tafenoquine or a comparator treatment.

Publication	Dose given (by descending dose)	Day	Total subjects	Keratopathy	Retinal changes	Visual field defects	Color vision changes	Visual acuity changes	Any ocular adverse effect
Nasveld, 2010^a^	TQ200/d loading, then 200 mg weekly × 26 weeks	week 26 ± 4	variable	69/74 (93%)^b^	27/69 (39%)	0/74	no difference between groups	no difference between groups	0/492
Nasveld, 2010^a^	MQ250/d loading then weekly × 26 weeks		variable	0/21 (0%)	4/17 (24%)	0/21			0/162
Warrasak, 2018	TQ400x3d	week 26 ± 4	44	14 (32%)	10 (22%)	7 (16%)	0/0^d^	0.31/0.31^e^	NR
Warrasak, 2018	CQ+PQ 15 mg/d		24	0	2 (8%)	3 (13%)	0/0^d^	0.31/0.31^e^	NR
Llanos-Cuentas, 2014	CQ+any TQ-50 to TQ-600	day 28, 90	61	7 (11%)	0	7 (11%)	NR	NR	NR
Llanos-Cuentas, 2014	CQ+PQ 15/d		15	1 (7%)	0	1 (7%)	NR	NR	NR
Llanos-Cuentas, 2014	CQ only		17	1 (6%)	0	1 (6%)	NR	NR	NR
Lacerda, 2019	CQ alone	day 29, 90	29	0	1 (3%)	1/24 (4%)	4 (14%)^d^	0	NR
Lacerda, 2019	CQ+TQ300		65	1 (2%)	2 (3%)	3/54 (6%)	8 (12%)^d^	4 (6%)	NR
Lacerda, 2019	CQ+PQ 15 m/d		31	0	1 (3%)	2/25 (8%)	3 (10%)^d^	0	NR
Llanos-Cuentas, 2019	CQ+TQ300	day 29, 90	27	0	0	5 (19%)	6 (22%)^d^	4 (15%)	NR^f^
Llanos-Cuentas, 2019	CQ+PQ 15 m/d		13	0	0	2 (15%)	0/0^d^	1 (8%)	NR^f^
Ackert, 2019	TQ300mg	day 90	330	1 (<1%)^c^	1 (<1%)	NR	NR	0.011/0.05^e^	9 (3%)
Ackert, 2019	Placebo		168	0	1 (<1%)	NR	NR	−0.004/−0.001^e^	7 (4%)

All data reported as n subjects examined (%) or if the total n is variable it is provided in the denominator. NR – not reported.

a This study does not have baseline ophthalmic measurements.

b Keratopathy resolved in 100% of subjects by 1 year.

c The same corneal changes were noted at baseline and day 90 in this subject.

d Color perception test is PIP. In this table, N represents subjects missing any number of plates.

e Visual acuity (expressed as LogMAR) reported as mean change at day 28 from baseline. A 0.08 LogMAR change represents a 1 line change on the Snellen visual acuity chart.

f Authors reported the ocular adverse events for an integrated analysis of 3 studies; Blurred vision in 5/483 (1%) TQ and 3/264 (1%) PQ groups overall.

## Article highlights

- Tafenoquine is a newly registered 8-aminoquinoline that is active against all stages of malaria. It can be used for causal prophylaxis or radical cure of *Plasmodium vivax*. There is no regulatory approval for the transmission-blocking, blood-stage treatment of any malaria, or presumptive anti-relapse treatment (PART) indications. However, the U.S. Centers for Disease Control and Prevention (CDC) malaria guidance has extended the use of tafenoquine for the radical cure to *P. ovale* and PART (Haston et al., 2019 [4]).
- Tafenoquine has a side effect profile similar to primaquine, which means generally it is well tolerated. However, 8-aminoquinolines including tafenoquine cause hemolysis in persons with intermediate and deficient G6PD activity. Psychosis is listed as a contraindication in the two approved tafenoquine labels (the U.S. Food and Drug Administration (FDA) and the Australian Therapeutic Goods Administration (TGA)) for causal prophylaxis while a history or current symptoms of psychiatric illness are a precaution in the radical cure labels with the same stringent regulatory agencies. Other less commonly known adverse effects have been reported.
- Twenty-four clinical trials reporting adverse events data comparing tafenoquine to a comparator drug or placebo arm were included in this systematic review.
- In the meta-analyses, no differences between tafenoquine and comparator groups were seen for neuropsychiatric, dermatologic, and most gastrointestinal adverse effects.
- In the five studies that assessed ophthalmologic outcomes, the clinical effects were mild and no different from the comparator.
- Tafenoquine monotherapy does not prolong the corrected QT interval.
- Precise comparisons of hemolysis were not possible due to methodologic heterogeneity. Although the comparison arm was favored, large hemoglobin reductions were not observed in G6PD-normal participants after tafenoquine use.
- Meta-analysis could not be performed for methemoglobin due to heterogenous reporting of results. Mild methemoglobin elevations were common. Clinically significant elevations were observed; however, they were uncommon.
- Pregnancy assessment and quantitative G6PD testing in the patient and breastfed infant, if applicable, are necessary before tafenoquine administration.
- Symptoms occurring up to 3 months after the last dose might be tafenoquine related due to its long plasma elimination half-life (12–17 days).
- In parts of South America, SE Asia, and Oceania, the radical curative efficacy of tafenoquine is low; in some areas both tafenoquine and low-dose primaquine have low efficacy. Further studies in these areas are needed to optimize the radical curative 8-aminoquinoline doses and select viable schizonticidal partner drugs and regimens for the anti-relapse treatment of *P. vivax* malaria. Continued safety assessments are needed to monitor tafenoquine toxicity. Although this should be balanced with the benefit of preventing morbidity from repeated *P. vivax* infections (e.g. anemia, hospitalization, low birth weight) and decreasing malaria transmission when efficacious tafenoquine regimens are used.

This box summarizes key points contained in the article.

## Drug summary box

Drug name (generic):Tafenoquine is marketed under Arakoda^™^ (USA) and Kodatef^®^ (Australia) for causal prophylaxis of malaria; and Krintafel^™^ (USA) and Kozenis^™^ (Australia) for radical cure of *Plasmodium vivax*

Phase: Tafenoquine has received regulatory approval in the USA and Australia. This article reviews animal studies (where there are few or no data in humans) and includes the Phase 1–3 human trials in a meta-analysis.

Indication: Treatment with tafenoquine is indicated for causal prophylaxis of all malarias, and radical cure for *P. vivax*. The US CDC issued guidance extending the use of radical cure tafenoquine to *P. ovale* and to presumptive anti-relapse treatment (PART).

Pharmacology description/mechanism of action: After oral administration, tafenoquine is absorbed slowly. Food may increase bioavailability. Maximum plasma concentrations of tafenoquine are reached between approximately 8–20 hours after drug administration (data from all the pharmacokinetic studies reviewed). Tafenoquine pharmacokinetics have been described adequately using either a one-or two-compartment model. The plasma elimination half-life is 12–17 days. The apparent volume of distribution is large (>2000L) indicating extensive distribution into tissue. More studies are needed to understand the metabolism of tafenoquine and the role of the liver isoenzyme CYP2D6 as reported for primaquine.

Route of administration: Only oral formulations are available.

Chemical structure:

Pivotal trials: As tafenoquine has only recently received regulatory approval (2018) nearly all of the trials included in this meta-analysis would be considered pivotal trials since they are the first in their field. Notably are the first in human trials for the characterization of pharmacokinetics (Brueckner RP, et al., 1998 [3]) and the first trials to establish doses for causal prophylaxis Shanks 2001 [8]) and radical cure Walsh 1999 [62] and Walsh 2004 [69] and [62]). Additionally, Edstein MD, et al., 2001 [28] & 2003 [30], Charles BG, et al., 2007 [27], Miller AK, et al., 2013 [34] and Thakkar N, et al., 2018 [29] provide pharmacokinetic data including the pharmacodynamics of tafenoquine efficacy and the association with adverse events. The dose related hemolytic effect of tafenoquine was first described in G6PD Mahidol heterozygous females with intermediate G6PD activity by Rueangweerayut R, et al., 2017 [7]). The Phase 2 trial establishing the radical cure dose (Llanos-Cuentas A, et al., 2014 [44]) and the Phase 3 trials (Lacerda MVG, et al., 2019 [43]; Llanos-Cuentas A, et al., 2019 [45]) demonstrating its safety, tolerability, and efficacy were critical for the regulatory approval of the radical cure indication.
