# Supplementary material for: Tafenoquine: a toxicity overview
Source: Expert Opin Drug Saf. Author manuscript; Available in PMC 2023 Oct 23. (PMC10591488; doi:10.1080/14740338.2021.1859476)
Supplement: Supplement [file NIHMS1883909-supplement-Supplement.pdf]

Supplement Table 1A. Data table used for the meta-analysis; the number of subjects reporting adverse symptoms

Publication	Drug	How AE assessed	Subjects	Psychiatric symptoms	Neurologic symptoms	Lethargy/ Weakness/ Fatigue	Headache	Dizziness/ Vertigo	Gastritis/ Abdominal pain	Nausea/ Vomiting	Diarrhea
Brueckner, 1998	placebo	periodic questioning	2	NA	NA	NA	2	1	0	0	0
	TQ-4		3	NA	NA	NA	0	0	0	0	3
	TQ-16		3	NA	NA	NA	0	0	0	0	0
	TQ-36		6	NA	NA	NA	0	1	0	0	0
	TQ-72		3	NA	NA	NA	0	0	0	0	0
	TQ-100		3	NA	NA	NA	0	0	0	0	0
	TQ-144		3	NA	NA	NA	0	0	0	0	0
	TQ-192		3	NA	NA	NA	0	0	0	0	0
	TQ-240		3	NA	NA	NA	0	0	0	0	0
	TQ-250		3	NA	NA	NA	0	0	0	0	0
	TQ-288		3	NA	NA	NA	1	0	0	0	0
	TQ-300		3	NA	NA	NA	1	0	2	0	0
	TQ-350		3	NA	NA	NA	0	2	0	0	0
	TQ-400		3	NA	NA	NA	0	0	0	0	0
	TQ-500		3	NA	NA	NA	0	0	0	0	1
	TQ-600		3	NA	NA	NA	0	0	2	1	0
Walsh, 1999	CQ	daily to day 14 after TQ dose completed	9	NA	NA	NA	NA	NA	NA	NA	NR
	TQ-300x7d		15	NA	NA	NA	NA	NA	NA	NA	2
	TQ-500x3d, and day 7		11	NA	NA	NA	NA	NA	NA	NA	NR
	TQ-500		9	NA	NA	NA	NA	NA	NA	NA	NR
Lell, 2000	placebo	weekly	82	NA	NA	0	2	0	1	NA	1
	TQ-31.25x3d		79	NA	NA	0	0	2	3	NA	0
	TQ-62.5x3d		86	NA	NA	1	10	3	4	NA	1
	TQ-125x3d		79	NA	NA	0	2	0	1	NA	1
	TQ-250x3d		84	NA	NA	1	5	0	7	NA	0
Shanks, 2001	placebo	weekly	61	NA	11	NA	11	NA	6	NA	7
	TQ-400x3d, placebo weekly		60	NA	11	NA	10	NA	5	NA	11
	TQ-200x3d, weekly		55	NA	14	NA	13	NA	4	NA	7
	TQ-400x3d, weekly		59	NA	15	NA	14	NA	11	NA	12
Nasveld, 2002	PQ-22.5x14d	NR	214	NA	NA	10	30	NA	9	32	2
	TQ-400x3d		292	NA	NA	12	88	NA	33	102	28
	TQ-200BDx3d		86	NA	NA	3	19	NA	11	20	13

All data reported as number of subjects (n) with study defined abnormal results.

NA - not mentioned if the data was collected, NR - data collected but raw indiv data not reported, d -days, w - weeks, m - months

Supplement Table 1A. Data table used for the meta-analysis; the number of subjects reporting adverse symptoms

Hale, 2003	placebo	days 1-14, then weekly	94	NA	NA	NA	6	NA	8	NA	15
	TQ-25x3d, weekly		93	NA	NA	NA	5	NA	5	NA	14
	TQ-50x3d, weekly		93	NA	NA	NA	7	NA	4	NA	14
	TQ-100x3d, weekly		94	NA	NA	NA	15	NA	11	NA	20
	TQ-200x3d, weekly		93	NA	NA	NA	4	NA	7	NA	12
	MFQ-250 weekly		46	NA	NA	NA	1	NA	4	NA	4
Walsh, 2004[1]	CQ+TQ-300x7d	daily during treatment	18	NA	8	8	4	8	3	1	1
	CQ+TQ-600x3d		19	NA	8	10	4	8	1	3	3
	CQ+TQ-600		18	NA	4	4	2	4	0	2	1
	CQ		13	NA	NA	NA	NA	NA	NA	NA	NA
	CQ+PQ-15x14d		12	NA	6	8	4	3	0	0	1
Walsh, 2004[2]	placebo	days 1-3, then after each dose	101	NA	NA	12	25	11	9	6	8
	TQ-400x3d, monthly x 5m		104	NA	NA	11	22	21	11	29	32
Elmes, 2008	PQ-7.5TIDx14d	day 3 and patient diary	464	NA	NA	8	9	NA	25	56	10
	TQ-400x3d		242	NA	NA	6	18	NA	56	73	23
	TQ-200BDx3d		161	NA	NA	3	5	NA	27	35	24
	TQ-200x3d		406	NA	NA	7	4	NA	28	34	19
Leary, 2009	placebo	eye test at weeks 3, 6, 12, 18, 24 and GFR weeks 12, 24	81	NA	NR	4	21	NR	NR	5	3
	TQ-200x3d, weekly		39	NA	NR	6	29	NR	NR	18	8
Nasveld, 2010	TQ-200x3d, weekly	baseline, then weeks 4, 8, 16, 26 and after PART weeks 2, 12	492	21	4	12	61	27	24	49	259
	MFQ-250x3d, weekly		162	6	1	6	20	10	13	21	81
Miller, 2013	CQ-1500+placebo	days 1-7 then days 10, 14, 28, 42, 56	20	NA	NA	NA	7	3	1	6	3
	TQ-450x2d+placebo		20	NA	NA	NA	3	2	1	8	3
	CQ-1500+TQ-450x2d		20	NA	NA	NA	7	6	3	11	4
Llanos-Cuentas, 2014	CQ+TQ-50	days 1-3, then days 5, 8, 11, 15, 22, 29, 60, 90, 180	55	2	5	NA	14	7	6	10	9
	CQ+TQ-100		57	3	4	NA	17	2	5	5	6
	CQ+TQ-300		57	5	1	NA	10	5	6	7	6
	CQ+TQ-600		56	3	5	NA	16	4	6	8	13
	CQ+PQ-15x14d		50	3	0	NA	14	5	7	9	6
	CQ		54	1	0	NA	20	5	5	3	6

All data reported as number of subjects (n) with study defined abnormal results.

NA - not mentioned if the data was collected, NR - data collected but raw indiv data not reported, d -days, w - weeks, m - months

Supplement Table 1A. Data table used for the meta-analysis; the number of subjects reporting adverse symptoms

Green, 2014	Placebo	Day -2, daily to day 6, then days 27, 63	50	NA	NA	NA	4	0	0	2	4
	TQ-300		48	NA	NA	NA	9	2	0	7	1
	TQ-600		52	NA	NA	NA	7	2	7	12	10
	TQ-400x3d		50	NA	NA	NA	8	2	3	18	5
	Moxiflox-200		51	NA	NA	NA	5	3	1	1	1
Green, 2016	DP+TQ-300	days 1-3, then days 7, 14, 21, 28, 56	24	NA	0	1	3	0	1	2	1
	AL+TQ-300		24	NA	1	1	4	2	1	1	1
	DP		24	NA	1	0	3	1	0	2	2
	AL		24	NA	0	1	1	0	0	0	0
	TQ-300		24	NA	0	0	1	0	0	0	0
Rueang-weerayut, 2017	TQ-100	days 1-14, then days 21, 28, 56	12	NR	NR	NR	NR	NR	NR	NR	NR
	TQ-200		12	NR	NR	NR	NR	NR	NR	NR	NR
	TQ-300		9	NR	NR	NR	NR	NR	NR	NR	NR
	PQ-15x14d		11	NR	NR	NR	NR	NR	NR	NR	NR
Fukuda, 2017	CQ+PQ-30x14d	days 1-28, then days 60, 90, 120	24	NR	4	NR	14	12	9	8	3
	TQ-400x3d		46	NR	2	NR	4	3	6	4	0
McCarthy, 2018	placebo	days 1-3, 10; phone follow up days 4-9, 11-12, 14-16	12	NA	1	1	4	NA	1	3	1
	TQ-800 (200x3d and day 10)		4	NA	0	0	4	NA	2	2	0
Lacerda, 2019	CQ+placebo	days 1-3, then days 5, 8, 11, 15, and days 22, 29, 60, 90, 120, 150, 180	133	4	4	2	19	11	24	21	9
	CQ+TQ-300		260	10	2	3	27	25	26	43	21
	CQ+PQ-15x14d		129	5	1	0	10	9	17	20	7
Llanos-Cuentas, 2019	CQ+TQ-300		166	5	2	3	27	29	12	42	12
	CQ+PQ-15x14d		85	5	4	0	16	16	2	15	3
Ackert, 2019	placebo	Days 7, 30, 60, 90	168	NA	NA	0	11	0	1	2	4
	TQ-300		330	NA	NA	3	23	2	1	19	4
Martin, 2003	placebo	Daily day -5 to -2 and 0-2, weekly for weeks 1-28	101	2	42	11	37	7	24	5	11
	TQ-200x3d, weekly x 24w		104	2	51	7	45	12	25	5	14
	TQ-200x3d, weekly x 24w		101	3	54	6	49	14	19	3	15
Edstein, 2006	TQ-400x3d	12 hours after the last dose	87	NA	NA	NA	NA	NA	30	37	14
	TQ-200BDx3d		86	NA	NA	NA	NA	NA	14	23	18

All data reported as number of subjects (n) with study defined abnormal results.

NA - not mentioned if the data was collected, NR - data collected but raw indiv data not reported, d -days, w - weeks, m - months

Supplement Table 1B. Data table used for the meta-analysis; the number of subjects reporting adverse symptoms

Publication	Drug	How AE assessed	Subjects	Fever	Myalgia	Rash/ Dermatologic	Eye irritation	Vision changes	SAE
Brueckner, 1998	placebo	periodic questioning	2	2	NA	1	0	NA	0
	TQ-4		3	0	NA	0	0	NA	0
	TQ-16		3	0	NA	0	1	NA	0
	TQ-36		6	0	NA	0	0	NA	0
	TQ-72		3	0	NA	0	0	NA	0
	TQ-100		3	0	NA	0	0	NA	0
	TQ-144		3	0	NA	0	0	NA	0
	TQ-192		3	0	NA	0	0	NA	0
	TQ-240		3	0	NA	0	0	NA	0
	TQ-250		3	0	NA	0	0	NA	0
	TQ-288		3	0	NA	0	0	NA	0
	TQ-300		3	0	NA	0	0	NA	0
	TQ-350		3	0	NA	0	0	NA	0
	TQ-400		3	0	NA	0	0	NA	0
	TQ-500		3	0	NA	0	0	NA	0
	TQ-600		3	0	NA	0	0	NA	0
Walsh, 1999	CQ	daily to day 14 after TQ dose completed	9	NA	NA	NA	NA	NA	0
	TQ-300x7d		15	NA	NA	NA	NA	NA	0
	TQ-500x3d, and day 7		11	NA	NA	NA	NA	NA	0
	TQ-500		9	NA	NA	NA	NA	NA	0
Lell, 2000	placebo	weekly	82	1	NA	2	NA	NA	0
	TQ-31.25x3d		79	0	NA	1	NA	NA	0
	TQ-62.5x3d		86	3	NA	0	NA	NA	0
	TQ-125x3d		79	1	NA	0	NA	NA	0
	TQ-250x3d		84	3	NA	0	NA	NA	0
Shanks, 2001	placebo	weekly	61	NA	12	6	NA	NA	0
	TQ-400x3d, placebo weekly		60	NA	11	13	NA	NA	0
	TQ-200x3d, weekly		55	NA	10	14	NA	NA	0
	TQ-400x3d, weekly		59	NA	11	16	NA	NA	2
Nasveld, 2002	PQ-22.5x14d	NR	214	NA	NA	NA	NA	NA	0
	TQ-400x3d		292	NA	NA	NA	NA	NA	0
	TQ-200BDx3d		86	NA	NA	NA	NA	NA	0

All data reported as number of subjects (n) with study defined abnormal results.
NA - not mentioned if the data was collected, NR - data collected but raw indiv data not reported, d -days, w - weeks, m - months

Supplement Table 1B. Data table used for the meta-analysis; the number of subjects reporting adverse symptoms

Hale, 2003	placebo	days 1-14, then weekly	94	NA	4	7	NA	NA	0
	TQ-25x3d, weekly		93	NA	9	0	NA	NA	0
	TQ-50x3d, weekly		93	NA	5	2	NA	NA	0
	TQ-100x3d, weekly		94	NA	12	3	NA	NA	0
	TQ-200x3d, weekly		93	NA	13	1	NA	NA	0
	MFQ-250 weekly		46	NA	3	1	NA	NA	0
Walsh, 2004[1]	CQ+TQ-300x7d	daily during treatment	18	NA	0	2	NA	NA	0
	CQ+TQ-600x3d		19	NA	1	1	NA	NA	0
	CQ+TQ-600		18	NA	0	1	NA	NA	0
	CQ		13	NA	NA	NA	NA	NA	0
	CQ+PQ-15x14d		12	NA	0	2	NA	NA	0
Walsh, 2004[2]	placebo	days 1-3, then after each dose	101	14	NA	6	NA	NA	6
	TQ-400x3d, monthly x 5m		104	9	NA	8	NA	NA	2
Elmes, 2008	PQ-7.5TIDx14d	day 3 and patient diary	464	NA	NA	NA	NA	NA	0
	TQ-400x3d		242	NA	NA	NA	NA	NA	1
	TQ-200BDx3d		161	NA	NA	NA	NA	NA	0
	TQ-200x3d		406	NA	NA	NA	NA	NA	0
Leary, 2009	placebo	eye test at weeks 3, 6, 12, 18, 24 and GFR weeks 12, 24	81	2	0	NA	NA	1	3
	TQ-200x3d, weekly		39	4	5	NA	NA	1	8
Nasveld, 2010	TQ-200x3d, weekly	baseline, then weeks 4, 8, 16,	492	NA	NA	70	NA	0	18
	MFQ-250x3d, weekly	26 and after PART weeks 2, 12	162	NA	NA	21	NA	0	3
Miller,2013	CQ-1500+placebo	days 1-7 then days 10, 14, 28, 42, 56	20	NA	NA	NA	NA	0	0
	TQ-450x2d+placebo		20	NA	NA	NA	NA	1	0
	CQ-1500+TQ-450x2d		20	NA	NA	NA	NA	0	0
Llanos-Cuentas, 2014	CQ+TQ-50	days 1-3, then days 5, 8, 11, 15, 22, 29, 60, 90, 180	55	18	3	4	NA	7	2
	CQ+TQ-100		57	16	4	8	NA		6
	CQ+TQ-300		57	5	1	8	NA		2
	CQ+TQ-600		56	7	3	2	NA		5
	CQ+PQ-15x14d		50	12	2	3	NA	1	7
	CQ		54	21	3	7	NA	1	4

All data reported as number of subjects (n) with study defined abnormal results.

NA - not mentioned if the data was collected, NR - data collected but raw indiv data not reported, d -days, w - weeks, m - months

Supplement Table 1B. Data table used for the meta-analysis; the number of subjects reporting adverse symptoms

Green, 2014	Placebo	Day -2, daily to day 6, then days 27, 63	50	NA	0	0	NA	NA	1
	TQ-300		48	NA	0	1	NA	NA	1
	TQ-600		52	NA	0	0	NA	NA	4
	TQ-400x3d		50	NA	0	0	NA	NA	0
	Moxiflox-200		51	NA	2	2	NA	NA	0
Green, 2016	DP+TQ-300	days 1-3, then days 7, 14, 21, 28, 56	24	3	NA	2	NA	NA	0
	AL+TQ-300		24	2	NA	0	NA	NA	0
	DP		24	2	NA	1	NA	NA	1
	AL		24	0	NA	1	NA	NA	1
	TQ-300		24	0	NA	0	NA	NA	0
Rueang-weerayut, 2017	TQ-100	days 1-14, then days 21, 28, 56	12	NR	NR	NR	NR	NR	0
	TQ-200		12	NR	NR	NR	NR	NR	0
	TQ-300		9	NR	NR	NR	NR	NR	0
	PQ-15x14d		11	NR	NR	NR	NR	NR	0
Fukuda, 2017	CQ+PQ-30x14d	days 1-28, then days 60, 90, 120	24	5	3	2	NR	NR	0
	TQ-400x3d		46	3	1	1	NR	NR	0
McCarthy, 2018	placebo	days 1-3, 10; phone follow up days 4-9, 11-12, 14-16	12	1	2	NA	NA	NA	0
	TQ-800 (200x3d and day 10)		4	0	2	NA	NA	NA	0
Lacerda, 2019	CQ+placebo	days 1-3, then days 5, 8, 11, 15, and days 22, 29, 60, 90, 120, 150, 180	133	2	19	21	NA	2	6
	CQ+TQ-300		260	9	15	36	NA	3	21
	CQ+PQ-15x14d		129	4	10	15	NA	1	4
Llanos-Cuentas, 2019	CQ+TQ-300		166	7	16	23	NA	3	2
	CQ+PQ-15x14d		85	6	11	20	NA	0	0
Ackert, 2019	placebo	Days 7, 30, 60, 90	168	NA	NA	3	0	7	0
	TQ-300		330	NA	NA	6	2	9	0
Martin, 2003	placebo	Daily day -5 to -2 and 0-2, weekly for weeks 1-28	101	2	25	13	13	0	2
	TQ-200x3d, weekly x 24w		104	0	27	13	12	1	4
	TQ-200x3d, weekly x 24w		101	1	29	11	7	1	1
Edstein, 2006	TQ-400x3d	12 hours after the last dose	87	NA	NA	NA	NA	NA	0
	TQ-200BDx3d		86	NA	NA	NA	NA	NA	0

All data reported as number of subjects (n) with study defined abnormal results.

NA - not mentioned if the data was collected, NR - data collected but raw indiv data not reported, d -days, w - weeks, m - months

Supplement Table 1C. Data table used for the meta-analysis; the number of subjects with abnormal (study defined) investigations

Publication	Drug	How AE assessed	Subjects	Methemo-globin	Hemolysis	Low platelets	WBC changes	High ALT	High creatinine	ECG changes
Brueckner, 1998	placebo	periodic questioning	2	0	0	0	0	NA	NA	0
	TQ-4		3	0	0	0	0	NA	NA	0
	TQ-16		3	0	0	0	0	NA	NA	0
	TQ-36		6	0	0	0	0	NA	NA	0
	TQ-72		3	0	0	0	0	NA	NA	0
	TQ-100		3	0	0	0	0	NA	NA	0
	TQ-144		3	0	0	0	0	NA	NA	0
	TQ-192		3	0	0	0	0	NA	NA	0
	TQ-240		3	0	0	0	0	NA	NA	0
	TQ-250		3	0	0	0	0	NA	NA	0
	TQ-288		3	0	0	0	0	NA	NA	0
	TQ-300		3	0	0	0	0	NA	NA	0
	TQ-350		3	0	0	0	0	NA	NA	0
	TQ-400		3	0	0	0	0	NA	NA	0
	TQ-500		3	0	0	0	0	NA	NA	0
	TQ-600		3	0	0	0	0	NA	NA	0
Walsh, 1999	CQ	daily to day 14 after TQ dose completed	9	<3	NA	NA	NA	NA	NA	NA
	TQ-300x7d		15	13.5	NA	NA	NA	NA	NA	NA
	TQ-500x3d, and day 7		11	14.7	NA	NA	NA	NA	NA	NA
	TQ-500		9	6.4	NA	NA	NA	NA	NA	NA
Lell, 2000	placebo	weekly	82	NA	NR	NR	NR	NR	NA	NA
	TQ-31.25x3d		79	NA	NR	NR	NR	NR	NA	NA
	TQ-62.5x3d		86	NA	NR	NR	NR	NR	NA	NA
	TQ-125x3d		79	NA	NR	NR	NR	NR	NA	NA
	TQ-250x3d		84	NA	NR	NR	NR	NR	NA	NA
Shanks, 2001	placebo	weekly	61	NR	NR	NR	NR	NR	NA	NA
	TQ-400x3d, placebo weekly		60	NR	NR	NR	NR	NR	NA	NA
	TQ-200x3d, weekly		55	NR	NR	NR	NR	NR	NA	NA
	TQ-400x3d, weekly		59	NR	NR	NR	NR	NR	NA	NA
Nasveld, 2002	PQ-22.5x14d	NR	214	NA	NA	NA	NA	NA	NA	NA
	TQ-400x3d		292	NA	NA	NA	NA	NA	NA	NA
	TQ-200BDx3d		86	NA	NA	NA	NA	NA	NA	NA

All data reported as number of subjects (n) with study defined abnormal results.

NA - not mentioned if the data was collected, NR - data collected but raw indiv data not reported, d -days, w - weeks, m - months

Supplement Table 1C. Data table used for the meta-analysis; the number of subjects with abnormal (study defined) investigations

Hale, 2003	placebo	days 1-14, then weekly	94	NA	1	0	NR	2	NA	NA
	TQ-25x3d, weekly		93	NA	2	0	NR	4	NA	NA
	TQ-50x3d, weekly		93	NA	2	0	NR	4	NA	NA
	TQ-100x3d, weekly		94	NA	0	0	NR	7	NA	NA
	TQ-200x3d, weekly		93	NA	3	0	NR	6	NA	NA
	MFQ-250 weekly		46	NA	0	0	NR	0	NA	NA
Walsh, 2004[1]	CQ+TQ-300x7d	daily during treatment	18	NR	NR	NR	NR	NR	NA	NA
	CQ+TQ-600x3d		19	NR	NR	NR	NR	NR	NA	NA
	CQ+TQ-600		18	NR	NR	NR	NR	NR	NA	NA
	CQ		13	NR	NR	NR	NR	NR	NA	NA
	CQ+PQ-15x14d		12	NR	NR	NR	NR	NR	NA	NA
Walsh, 2004[2]	placebo	days 1-3, then after each dose	101	NR	NR	NR	NR	NA	NA	NA
	TQ-400x3d, monthly x 5m		104	23	NR	NR	NR	12	NA	NA
Elmes, 2008	PQ-7.5TIDx14d	day 3 and patient diary	464	NA	NA	NA	NA	NA	NA	NA
	TQ-400x3d		242	NA	NA	NA	NA	NA	NA	NA
	TQ-200BDx3d		161	NA	NA	NA	NA	NA	NA	NA
	TQ-200x3d		406	NA	NA	NA	NA	NA	NA	NA
Leary, 2009	placebo	eye test at weeks 3, 6, 12, 18, 24 and GFR weeks 12, 24	81	NA	0	NR	NR	NA	1	NR
	TQ-200x3d, weekly		39	NA	1	NR	NR	NA	3	NR
Nasveld, 2010	TQ-200x3d, weekly	baseline, then weeks 4, 8, 16, 26 and after PART weeks 2, 12	492	NA	2	NR	NR	NA	93	0
	MFQ-250x3d, weekly		162	NA	0	NR	NR	NA	16	0
Miller,2013	CQ-1500+placebo	days 1-7 then days 10, 14, 28, 42, 56	20	0	1	NA	NA	NA	NA	0
	TQ-450x2d+placebo		20	0	4	NA	NA	NA	NA	0
	CQ-1500+TQ-450x2d		20	3	3	NA	NA	NA	NA	0
Llanos-Cuentas, 2014	CQ+TQ-50	days 1-3, then days 5, 8, 11, 15, 22, 29, 60, 90, 180	55	0	0	NR	NR	1	NR	3
	CQ+TQ-100		57	0	1	NR	NR	1	NR	2
	CQ+TQ-300		57	0	2	NR	NR	4	NR	3
	CQ+TQ-600		56	0	1	NR	NR	3	NR	1
	CQ+PQ-15x14d		50	1	1	NR	NR	4	NR	5
	CQ		54	0	1	NR	NR	1	NR	4

All data reported as number of subjects (n) with study defined abnormal results.

NA - not mentioned if the data was collected, NR - data collected but raw indiv data not reported, d -days, w - weeks, m - months

Supplement Table 1C. Data table used for the meta-analysis; the number of subjects with abnormal (study defined) investigations

Green, 2014	Placebo	Day -2, daily to day 6, then days 27, 63	50	NR	NR	NR	NR	NR	NR	0
	TQ-300		48	NR	NR	NR	NR	2	NR	0
	TQ-600		52	NR	NR	NR	NR	1	NR	0
	TQ-400x3d		50	NR	NR	NR	NR	2	NR	0
	Moxiflox-200		51	NR	NR	NR	NR	NR	NR	1
Green, 2016	DP+TQ-300	days 1-3, then days 7, 14, 21, 28, 56	24		0	0	0	0	0	0
	AL+TQ-300		24		0	0	0	0	0	0
	DP		24		0	0	0	0	0	1
	AL		24		0	0	0	2	0	1
	TQ-300		24		0	0	0	0	0	0
Rueang-weerayut, 2017	TQ-100	days 1-14, then days 21, 28, 56	12	4	0	NR	NR	NR	NR	NR
	TQ-200		12	0	1	NR	NR	NR	NR	NR
	TQ-300		9	3	3	NR	NR	NR	NR	NR
	PQ-15x14d		11	8	2	NR	NR	NR	NR	NR
Fukuda, 2017	CQ+PQ-30x14d	days 1-28, then days 60, 90, 120	24	22	NR	6	0	2	NR	NR
	TQ-400x3d		46	0	NR	0	0	0	NR	NR
McCarthy, 2018	placebo	days 1-3, 10; phone follow up days 4-9, 11-12, 14-16	12	NA	0	NR	NR	NR	NR	NA
	TQ-800 (200x3d and day 10)		4	NA	2	NR	NR	NR	NR	NA
Lacerda, 2019	CQ+placebo	days 1-3, then days 5, 8, 11, 15, and days 22, 29, 60, 90, 120, 150, 180	133	0	2	14	0	8	0	3
	CQ+TQ-300		260	0	14	35	3	6	1	0
	CQ+PQ-15x14d		129	2	2	15	2	3	0	0
Llanos-Cuentas,	CQ+TQ-300		166	0	4	1	1	8	0	5
	CQ+PQ-15x14d		85	1	1	0	0	0	0	2
Ackert, 2019	placebo	Days 7, 30, 60, 90	168	NA	NA	NA	NA	0	NA	NA
	TQ-300		330	NA	NA	NA	NA	1	NA	NA
Martin, 2003	placebo	Daily day -5 to -2 and 0-2, weekly for weeks 1-28	101	NA	0	10/82	6	0/82	4	NA
	TQ-200x3d, weekly x 24w		104	NA	1	15/85	9	0/85	16	NA
	TQ-200x3d, weekly x 24w		101	NA	0	8/88	9	1/88	19	NA
Edstein, 2006	TQ-400x3d	12 hours after the last dose	87	NA	NA	NA	NA	NA	NA	NA
	TQ-200BDx3d		86	NA	NA	NA	NA	NA	NA	NA

All data reported as number of subjects (n) with study defined abnormal results.

NA - not mentioned if the data was collected, NR - data collected but raw indiv data not reported, d -days, w - weeks, m - months

Supplement Table 2. Methemoglobin data after tafenoquine or comparison drug regimens

Publication	Drug group	N	Absolute Methb maximum (%) reported unless otherwise indicated
Walsh, DS et al., 1998	CQ	9	<3
Walsh, DS et al., 1998	TQ-2100 (300x7d)	15	13.5
Walsh, DS et al., 1998	TQ-2000 (500x3d, and d7)	11	14.7
Walsh, DS et al., 1998	TQ-500	9	6.4
Shanks GD, et al., 2001	placebo	61	Not reported
Shanks GD, et al., 2001	TQ-1200 (400x3d), placebo weekly	60	Not reported
Shanks GD, et al., 2001	TQ-1200 (200x3d), weekly	55	Mean (SD); 2.5 (1.6)
Shanks GD, et al., 2001	TQ-1200 (400x3d), weekly	59	Mean (SD); 4.5 (2.5)
Walsh, DS et al., 2004[1]	CQ+TQ-300x7d	18	Mean maximum; 12.1
Walsh, DS et al., 2004[1]	CQ+TQ-600x3d	19	Mean maximum; 9
Walsh, DS et al., 2004[1]	CQ+TQ-600	18	Mean maximum; 4.5
Walsh, DS et al., 2004[1]	CQ	13	Mean maximum; 1.1
Walsh, DS et al., 2004[1]	CQ+PQ-15x14d	12	Mean maximum; 3.3
Walsh, DS et al., 2004[2]	placebo	101	Not reported
Walsh, DS et al., 2004[2]	TQ-400x3d, monthlyx5m	104	6.9
Walsh, DS et al., 2004[2]	TQ-400x3d, weekly	10	9.8
Nasveld, P et al., 2010	TQ-200x3d, weekly	492	Absolute change from mean; 1.8
Nasveld, P et al., 2010	MFQ-250x3d, weekly	162	Absolute change from mean; 0.1
Miller, A et al., 2013	CQ-1500+placebo	20	Mean maximum; 0.5
Miller, A et al., 2013	TQ-450x2d+placebo	20	Mean maximum; 4.0
Miller, A et al., 2013	CQ-1500+TQ-450x2d	20	Mean maximum; 6.2
Green JA, et al., 2014	Placebo	50	Not reported
Green JA, et al., 2014	TQ-300	48	Mean maximum change (SD); 0.2 (0.5)
Green JA, et al., 2014	TQ-600	52	Mean maximum change (SD); 1.1 (1.2)
Green JA, et al., 2014	TQ-1200 (400x3d)	50	12.2
Green JA, et al., 2014	Moxiflox-200	51	Not reported
Green JA, et al. 2016	DP+TQ-300	24	Mean (SD); 1.3 ([a])
Green JA, et al. 2016	AL+TQ-300	24	Mean (SD); 1.4 ([a])
Green JA, et al. 2016	DP	24	Mean (SD); 1.2 ([a])
Green JA, et al. 2016	AL	24	Mean (SD); 1.1 ([a])
Green JA, et al. 2016	TQ-300	24	Mean (SD); 1.5 ([a])
Rueangweerayut, R et al., 2017	TQ-100	12	5
Rueangweerayut, R et al., 2017	TQ-200	12	2
Rueangweerayut, R et al., 2017	TQ-300	9	5
Rueangweerayut, R et al., 2017	PQ-15x14d	11	13.1
Fukuda M, et al., 2017	CQ+PQ-30x14d	24	5.9
Fukuda M, et al., 2017	TQ-1200 (400x3d)	46	25.6
Lacerda, M, et al. 2019	CQ+placebo	133	Not reported
Lacerda, M, et al. 2019	CQ+TQ-300	260	13
Lacerda, M, et al. 2019	CQ+PQ-15x14d	129	14.7

Numeric superscript indicates multiple publications by the same first author in the same calendar year.
[a] Standard deviation was plotted on the figure in the manuscript, but the value could not be determined due to the overlapping lines.

Supplement Figure 1. Relative risk for headache with tafenoquine as compared to placebo or a control group

Group by Strata	Study name	Comparison	Risk ratio	Lower limit	Upper limit	p-Value
1. Placebo	Brueckner 1998	Placebo vs. TQ-300	2.22	0.57	8.68	0.25
1. Placebo	Green 2014	Placebo vs. TQ-300	0.43	0.14	1.29	0.13
1. Placebo	Ackert 2019	Placebo vs. TQ-300	0.94	0.47	1.88	0.86
1. Placebo	Lell 2000	Placebo vs. TQ-750	0.41	0.08	2.05	0.28
1. Placebo	McCarthy 2018	Placebo vs. TQ-800	0.38	0.17	0.86	0.02
1. Placebo	Hale 2003-1	Placebo vs. TQ-3000	1.48	0.43	5.09	0.53
1. Placebo	Shanks 2001	Placebo vs. TQ-3200	0.76	0.37	1.56	0.46
1. Placebo	Walsh 2004b	Placebo vs. TQ-3200	1.17	0.71	1.94	0.54
1. Placebo	Martin 2003-1	Placebo vs. TQ-5400	0.85	0.60	1.19	0.33
1. Placebo	Leary 2009	Placebo vs. TQ-5600	0.35	0.23	0.53	0.00
1. Placebo			0.72	0.49	1.08	0.11
2. ACT/CQ	Green 2016-1	AL vs. AL+TQ-300	0.25	0.03	2.08	0.20
2. ACT/CQ	Green 2016-2	DP vs. DP+TQ-300	1.00	0.22	4.47	1.00
2. ACT/CQ	Llanos-Cuentas 2014-1	CQ vs. CQ+TQ-300	2.11	1.09	4.09	0.03
2. ACT/CQ	Lacerda 2019-1	CQ vs. CQ+TQ-300	1.38	0.79	2.38	0.25
2. ACT/CQ	Miller 2013	CQ vs. CQ+TQ-900	1.00	0.43	2.33	1.00
2. ACT/CQ			1.23	0.67	2.27	0.51
3. PQ	Llanos-Cuentas 2014-2	CQ+PQ vs. CQ+TQ-300	1.60	0.78	3.27	0.20
3. PQ	Lacerda 2019-2	CQ+PQ vs. CQ+TQ-300	0.75	0.37	1.49	0.41
3. PQ	Llanos-Cuentas 2019	CQ+PQ vs. CQ+TQ-300	1.16	0.66	2.03	0.61
3. PQ	Walsh 2004a	CQ+PQ vs. CQ+TQ-600	3.00	0.65	13.88	0.16
3. PQ	Elmes 2008	PQ vs. TQ-600	1.97	0.61	6.34	0.26
3. PQ	Fukuda 2017	CQ+PQ vs. TQ-1200	6.71	2.48	18.15	0.00
3. PQ	Nasveld 2002	PQ vs. TQ-1200	0.50	0.34	0.72	0.00
3. PQ			1.31	0.81	2.11	0.26
4. MQ	Hale 2003-2	MQ vs. TQ-3000	0.51	0.06	4.39	0.54
4. MQ	Martin 2003-2	MQ vs. TQ-5400	1.12	0.83	1.51	0.45
4. MQ	Nasveld 2010	MQ vs. TQ-5800	1.00	0.62	1.60	0.99
4. MQ			0.99	0.49	2.00	0.99

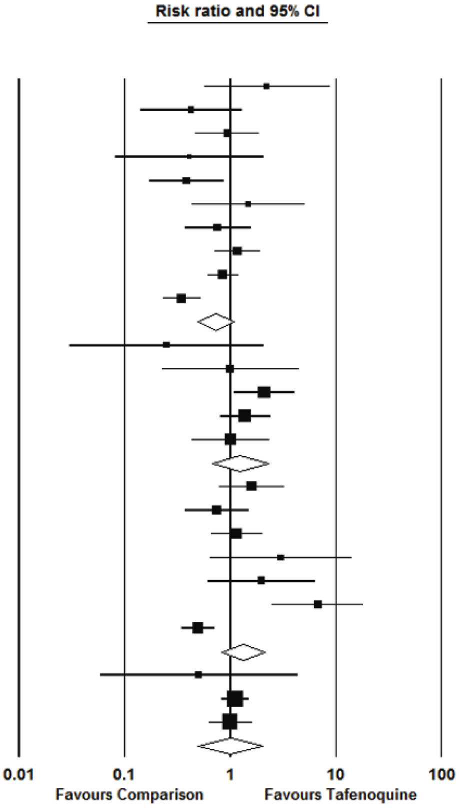

Supplement Figure 2. Relative risk for dizziness with tafenoquine as compared to placebo or a control group

Group by Strata	Study name	Comparison	Risk ratio	Lower limit	Upper limit	p-Value
1. Placebo	Brueckner 1998	Placebo vs. TQ-300	4.00	0.24	67.71	0.34
1. Placebo	Green 2014	Placebo vs. TQ-300	0.19	0.01	3.90	0.28
1. Placebo	Ackert 2019	Placebo vs. TQ-300	0.39	0.02	8.11	0.54
1. Placebo	Walsh 2004b	Placebo vs. TQ-3200	0.54	0.27	1.06	0.07
1. Placebo	Martin 2003-1	Placebo vs. TQ-5400	0.60	0.25	1.46	0.26
1. Placebo			0.59	0.29	1.19	0.14
2. ACT/CQ	Green 2016-1	AL vs. AL+TQ-300	0.20	0.01	3.96	0.29
2. ACT/CQ	Green 2016-2	DP vs. DP+TQ-300	3.00	0.13	70.16	0.49
2. ACT/CQ	Llanos-Cuentas 2014-1	CQ vs. CQ+TQ-300	1.06	0.32	3.44	0.93
2. ACT/CQ	Lacerda 2019-1	CQ vs. CQ+TQ-300	0.86	0.44	1.69	0.66
2. ACT/CQ	Miller 2013	CQ vs. CQ+TQ-900	0.50	0.14	1.73	0.27
2. ACT/CQ			0.79	0.40	1.56	0.50
3. PQ	Llanos-Cuentas 2014-2	CQ+PQ vs. CQ+TQ-300	1.14	0.35	3.71	0.83
3. PQ	Lacerda 2019-2	CQ+PQ vs. CQ+TQ-300	0.73	0.35	1.51	0.39
3. PQ	Llanos-Cuentas 2019	CQ+PQ vs. CQ+TQ-300	1.08	0.62	1.87	0.79
3. PQ	Walsh 2004a	CQ+PQ vs. CQ+TQ-600	1.13	0.30	4.16	0.86
3. PQ	Fukuda 2017	CQ+PQ vs. TQ-1200	7.67	2.39	24.58	0.00
3. PQ			1.32	0.77	2.26	0.31
4. MQ	Martin 2003-2	MQ vs. TQ-5400	1.20	0.58	2.47	0.62
4. MQ	Nasveld 2010	MQ vs. TQ-5800	2.99	1.52	5.87	0.00
4. MQ			1.92	0.92	4.00	0.08

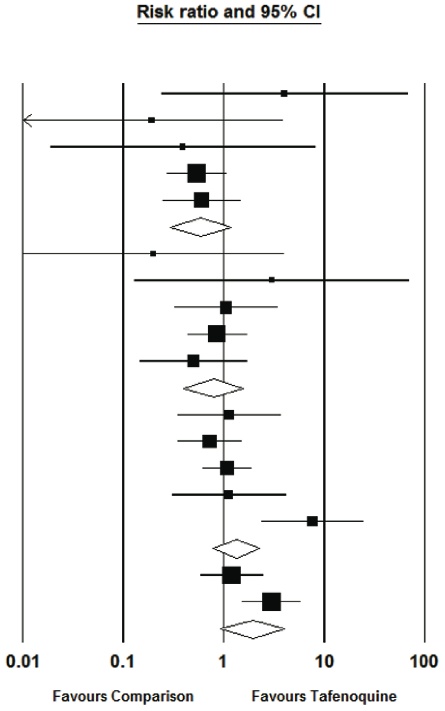

The reference for Martin 2003 ('study 030') is included in Novitt-Moreno A, et al., 2017. Details for the meta-analysis were obtained from the final clinical report by Martin et al., 2003. This footnote applies to all Supplement Figures, except Supplement Figure 4.

Supplement Figure 3. Relative risk for lethargy, weakness, or fatigue with tafenoquine as compared to placebo or a control group

Group by Strata	Study name	Comparison	Risk ratio	Lower limit	Upper limit	p-Value
1. Placebo	Ackert 2019	Placebo vs. TQ-300	0.28	0.01	5.39	0.40
1. Placebo	Martin 2003-1	Placebo vs. TQ-5400	2.32	0.74	7.28	0.15
1. Placebo	Lell 2000	Placebo vs. TQ-750	0.34	0.01	8.26	0.51
1. Placebo	McCarthy 2018	Placebo vs. TQ-800	1.15	0.06	23.88	0.93
1. Placebo	Walsh 2004b	Placebo vs. TQ-3200	1.12	0.52	2.43	0.77
1. Placebo	Leary 2009	Placebo vs. TQ-5600	0.32	0.10	1.07	0.06
1. Placebo			0.92	0.50	1.70	0.79
2. ACT/CQ	Green 2016-1	AL vs. AL+TQ-300	1.00	0.07	15.08	1.00
2. ACT/CQ	Green 2016-2	DP vs. DP+TQ-300	0.33	0.01	7.80	0.49
2. ACT/CQ	Lacerda 2019-1	CQ vs. CQ+TQ-300	1.30	0.22	7.70	0.77
2. ACT/CQ			0.94	0.23	3.78	0.93
3. PQ	Elmes 2008	PQ vs. TQ-600	1.00	0.37	2.73	1.00
3. PQ	Nasveld 2002	PQ vs. TQ-1200	1.18	0.54	2.57	0.68
3. PQ	Walsh 2004a	CQ+PQ vs. CQ+TQ-600	3.00	1.16	7.78	0.02
3. PQ	Lacerda 2019-2	CQ+PQ vs. CQ+TQ-300	0.29	0.01	5.51	0.41
3. PQ	Llanos-Cuentas 2019	CQ+PQ vs. CQ+TQ-300	0.28	0.01	5.31	0.39
3. PQ			1.32	0.74	2.37	0.35
4. MQ	Martin 2003-2	MQ vs. TQ-5400	0.51	0.10	2.75	0.44
4. MQ	Nasveld 2010	MQ vs. TQ-5800	1.52	0.58	3.98	0.40
4. MQ			1.12	0.44	2.85	0.82

Statistics for each study

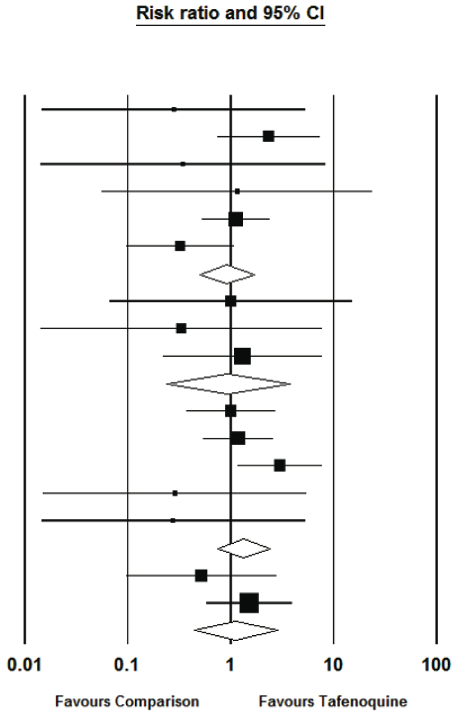

Supplement Figure 4. Relative risk for prolonged corrected QT interval with tafenoquine as compared to placebo or a control group

Group by Strata	Study name	Comparison	Risk ratio	Lower limit	Upper limit	p-Value
1. ACT/CQ	Green 2016-1	AL vs. AL+TQ-300	3.00	0.13	70.16	0.49
1. ACT/CQ	Green 2016-2	DP vs. DP+TQ-300	3.00	0.13	70.16	0.49
1. ACT/CQ	Lacerda 2019-1	CQ vs. CQ+TQ-300	13.63	0.71	262.03	0.08
1. ACT/CQ	Llanos-Cuentas 2014-1	CQ vs. CQ+TQ-300	1.41	0.33	6.00	0.64
1. ACT/CQ			2.37	0.77	7.29	0.13
2. PQ	Llanos-Cuentas 2014-2	CQ+PQ vs. CQ+TQ-300	1.90	0.48	7.55	0.36
2. PQ	Llanos-Cuentas 2019	CQ+PQ vs. CQ+TQ-300	0.78	0.15	3.94	0.76
2. PQ			1.31	0.46	3.74	0.62
Overall			1.72	0.80	3.72	0.16

Statistics for each study

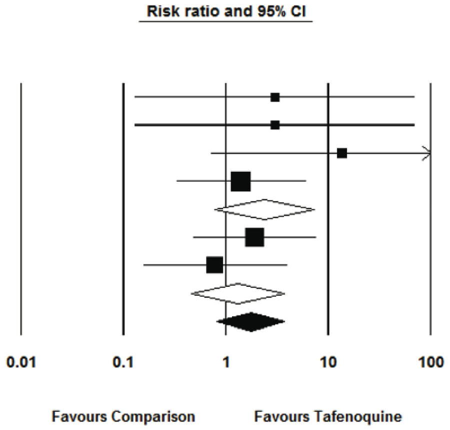

The reference for Martin 2003 ('study 030') is included in Novitt-Moreno A, et al., 2017. Details for the meta-analysis were obtained from the final clinical report by Martin et al., 2003. This footnote applies to all Supplement Figures, except Supplement Figure 4.

Supplement Figure 5. Relative risk for gastritis or abdominal pain with tafenoquine as compared to placebo or a control group

Group by Strata	Study name	Comparison	Risk ratio	Lower limit	Upper limit	p-Value
1. Placebo	Ackert 2019	Placebo vs. TQ-300	1.96	0.12	31.21	0.63
1. Placebo	Lell 2000	Placebo vs. TQ-750	0.15	0.02	1.16	0.07
1. Placebo	McCarthy 2018	Placebo vs. TQ-800	0.17	0.02	1.38	0.10
1. Placebo	Hale 2003-1	Placebo vs. TQ-3000	1.13	0.43	2.99	0.80
1. Placebo	Shanks 2001	Placebo vs. TQ-3200	1.35	0.40	4.54	0.63
1. Placebo	Walsh 2004b	Placebo vs. TQ-3200	0.84	0.36	1.95	0.69
1. Placebo	Martin 2003-1	Placebo vs. TQ-5400	0.99	0.61	1.61	0.96
1. Placebo			0.81	0.47	1.38	0.43
2. ACT/CQ	Green 2016-1	AL vs. AL+TQ-300	0.33	0.01	7.80	0.49
2. ACT/CQ	Green 2016-2	DP vs. DP+TQ-300	0.33	0.01	7.80	0.49
2. ACT/CQ	Lacerda 2019-1	CQ vs. CQ-TQ-300	1.80	1.08	3.02	0.02
2. ACT/CQ	Llanos-Cuentas 2014-1	CQ vs. CQ+TQ-300	0.88	0.29	2.71	0.82
2. ACT/CQ	Miller 2013	CQ vs. CQ+TQ-900	0.33	0.04	2.94	0.32
2. ACT/CQ			1.07	0.52	2.21	0.86
3. PQ	Llanos-Cuentas 2014-2	CQ+PQ vs. CQ+TQ-300	1.33	0.48	3.70	0.58
3. PQ	Lacerda 2019-2	CQ+PQ vs. CQ+TQ-300	1.32	0.74	2.34	0.35
3. PQ	Llanos-Cuentas 2019	CQ+PQ vs. CQ+TQ-300	0.33	0.07	1.42	0.14
3. PQ	Elmes 2008	PQ vs. TQ-600	0.78	0.46	1.32	0.35
3. PQ	Fukuda 2017	CQ+PQ vs. TQ-1200	2.88	1.16	7.12	0.02
3. PQ	Nasveld 2002	PQ vs. TQ-1200	0.36	0.18	0.73	0.00
3. PQ			0.92	0.57	1.48	0.73
4. MQ	Hale 2003-2	MQ vs. TQ-3000	1.16	0.36	3.75	0.81
4. MQ	Martin 2003-2	MQ vs. TQ-5400	0.78	0.46	1.33	0.36
4. MQ	Nasveld 2010	MQ vs. TQ-5800	1.65	0.86	3.15	0.13
4. MQ			1.12	0.58	2.14	0.74

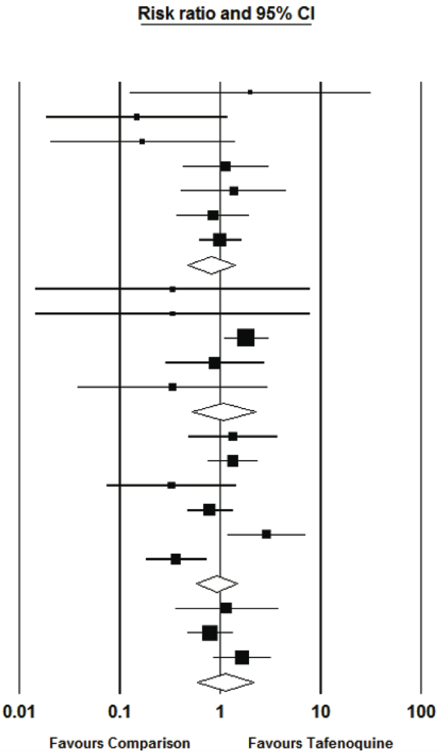

Supplement Figure 6. Relative risk for diarrhea with tafenoquine as compared to placebo or a control group

Group by Strata	Study name	Comparison	Risk ratio	Lower limit	Upper limit	p-Value
1. Placebo	Green 2014	Placebo vs. TQ-300	3.84	0.44	33.14	0.22
1. Placebo	Ackert 2019	Placebo vs. TQ-300	1.96	0.50	7.76	0.34
1. Placebo	Lell 2000	Placebo vs. TQ-750	3.07	0.13	74.34	0.49
1. Placebo	McCarthy 2018	Placebo vs. TQ-800	1.15	0.06	23.88	0.93
1. Placebo	Hale 2003-1	Placebo vs. TQ-3000	1.24	0.61	2.50	0.55
1. Placebo	Shanks 2001	Placebo vs. TQ-3200	0.90	0.34	2.41	0.84
1. Placebo	Walsh 2004b	Placebo vs. TQ-3200	0.26	0.12	0.53	0.00
1. Placebo	Martin 2003-1	Placebo vs. TQ-5400	0.81	0.39	1.70	0.58
1. Placebo	Leary 2009	Placebo vs. TQ-5600	0.18	0.05	0.64	0.01
1. Placebo			0.75	0.45	1.24	0.26
2. ACT/CQ	Green 2016-1	AL vs. AL+TQ-300	0.33	0.01	7.80	0.49
2. ACT/CQ	Green 2016-2	DP vs. DP+TQ-300	2.00	0.19	20.61	0.56
2. ACT/CQ	Lacerda 2019-1	CQ vs. CQ-TQ-300	0.84	0.39	1.78	0.64
2. ACT/CQ	Llanos-Cuentas 2014-1	CQ vs. CQ+TQ-300	1.06	0.36	3.07	0.92
2. ACT/CQ	Miller 2013	CQ vs. CQ+TQ-900	0.75	0.19	2.93	0.68
2. ACT/CQ			0.90	0.43	1.87	0.77
3. PQ	Llanos-Cuentas 2014-2	CQ+PQ vs. CQ+TQ-300	1.14	0.39	3.31	0.81
3. PQ	Lacerda 2019-2	CQ+PQ vs. CQ+TQ-300	0.67	0.29	1.54	0.35
3. PQ	Llanos-Cuentas 2019	CQ+PQ vs. CQ+TQ-300	0.49	0.14	1.68	0.26
3. PQ	Walsh 2004a	CQ+PQ vs. CQ+TQ-600	1.38	0.10	20.16	0.81
3. PQ	Elmes 2008	PQ vs. TQ-600	0.46	0.22	0.98	0.04
3. PQ	Fukuda 2017	CQ+PQ vs TQ-1200	13.16	0.71	244.78	0.08
3. PQ	Nasveld 2002	PQ vs. TQ-1200	0.09	0.02	0.35	0.00
3. PQ			0.57	0.32	1.03	0.06
4. MQ	Hale 2003-2	MQ vs. TQ-3000	0.67	0.23	1.97	0.47
4. MQ	Martin 2003-2	MQ vs. TQ-5400	1.10	0.56	2.17	0.78
4. MQ	Nasveld 2010	MQ vs. TQ-5800	0.95	0.80	1.13	0.56
4. MQ			0.93	0.49	1.75	0.82
Overall			0.76	0.55	1.05	0.09

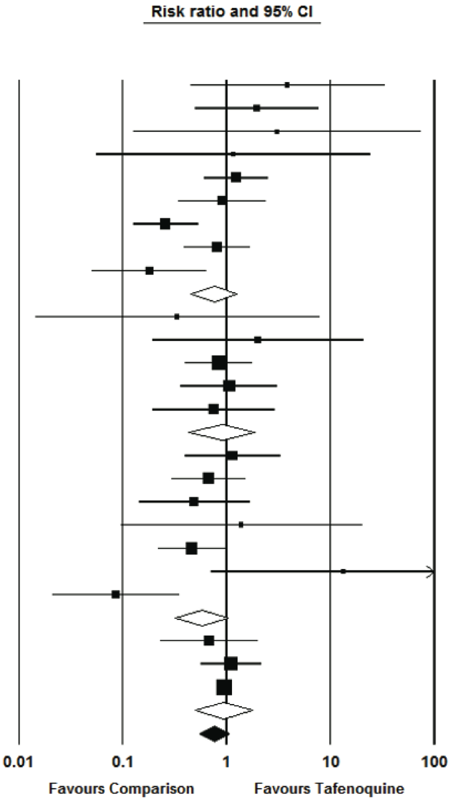

The reference for Martin 2003 ('study 030') is included in Novitt-Moreno A, et al., 2017. Details for the meta-analysis were obtained from the final clinical report by Martin et al., 2003. This footnote applies to all Supplement Figures, except Supplement Figure 4.
